# Programming Hydrogel Mechanics via Sequence-Controlled Polymerization Using Peptide Self-Assembly

**DOI:** 10.1021/jacs.5c12182

**Published:** 2026-01-29

**Authors:** Abolfazl S. Moghaddam, Maahi Zaman, Sz-Chian Liou, E. Thomas Pashuck

**Affiliations:** † Department of Bioengineering, 1687 Lehigh University, Bethlehem, Pennsylvania 18015, United States; ‡ Institute for Functional Materials and Devices, 1687 Lehigh University, Bethlehem, Pennsylvania 18015, United States

## Abstract

Hydrogels often have poor mechanical properties due to their high water content and low polymer concentration, which limits their utility in applications that require them to withstand applied forces. Inspired by natural biopolymers such as collagen and actin, which form highly extended fibrillar networks that stiffen biological tissues, we developed a modular strategy that utilizes self-assembling peptides to direct the formation of covalently polymerized diacetylene networks in hydrogels. By systematically tuning peptide sequences, we precisely controlled the supramolecular organization and molecular orientation within the self-assembled nanofibers. This optimization enabled efficient topotactic polymerization of diacetylene moieties within the self-assembling peptides. Peptide sequences that readily promoted polymerization formed hydrogels with superior viscoelastic properties. Incorporation of these diacetylene peptide amphiphiles (DA-PAs) into covalently cross-linked poly­(ethylene glycol) (PEG) hydrogels increased their mechanical stiffness 200-fold, while increasing viscous dissipation over 1,000 times. Modifying the chemical structure of the PEG cross-linker tuned the interfacial interactions between the covalent PEG and DA-PA networks, modulating stiffness by almost an order of magnitude. Since the DA-PAs readily dissolve in water prior to polymerization, they can be incorporated into most hydrogel systems. Adding them to alginate hydrogels led to an almost 20-fold increase in the hydrogel stiffness. This approach, merging peptide-driven supramolecular chemistry with precise covalent polymerization, provides powerful and versatile pathways for fabricating mechanically robust materials that offer new insights into how hierarchical structures can be used to improve hydrogel mechanics.

## Graphical abstract

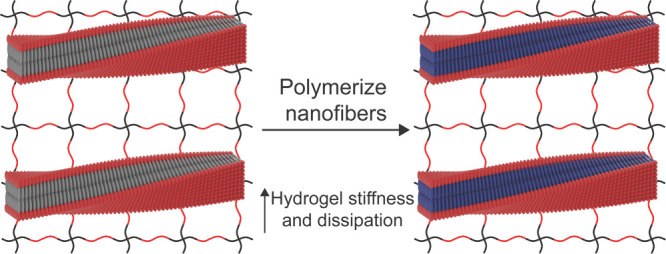

## Introduction

Hydrogels are highly hydrated polymer networks that constitute the primary structural component of most human tissues.^1,2^ These materials have important roles across diverse applications, including drug delivery,^3^ tissue modeling,^4^ wound dressings,^5^ sensing,^6^ soft robotics,^7^ and adhesives.^8^ Since water itself cannot sustain applied forces, the mechanical properties of hydrogels depend on the polymeric networks, which typically occupy only a small weight fraction. Consequently, many hydrogels exhibit inherently low stiffness and toughness relative to those of other classes of materials. These limited mechanical properties constrain the broader application of hydrogels in demanding load-bearing contexts, such as cartilage replacement,^9^ vascular grafts,^10^ flexible bioelectronic sensors,^11^ and injectable hydrogels.^12^ This has made the development of novel strategies to enhance the mechanical performance of hydrogels a highly active area of research.^13^

In biological tissues, cells are embedded within a fibrous extracellular matrix (ECM). The mechanical properties of the ECM are largely due to collagen, the most abundant protein in mammals (Figure 1A),^14,15^ while the mechanical integrity of cells primarily arises from filamentous cytoskeletal proteins, notably actin (Figure 1B).^16^ Collagen forms extended fibrillar structures essential for providing strength, resisting deformation, and transmitting mechanical forces efficiently over large distances.^17,18^ Individual tropocollagen molecules are helical with lengths of approximately 280 nm. These highly extended structures maximize intermolecular interactions, which are a primary driver of the high rigidity and robust mechanical properties of collagens.^18^ The formation of such long, stiff protein nanostructures relies upon highly directional molecular interactions and organized molecular packing.^19–21^ Nature achieves remarkable mechanical properties in tissues through the precise molecular control and alignment of large biopolymers. However, synthetic replication of these extended protein networks remains challenging as synthetic polymers commonly adopt random coil configurations. A promising alternative strategy involves the use of molecules designed to promote directional supramolecular interactions, such as hydrogen bonding, to form high aspect ratio nanostructures that template subsequent covalent polymerization.^22–25^

**Figure 1:**
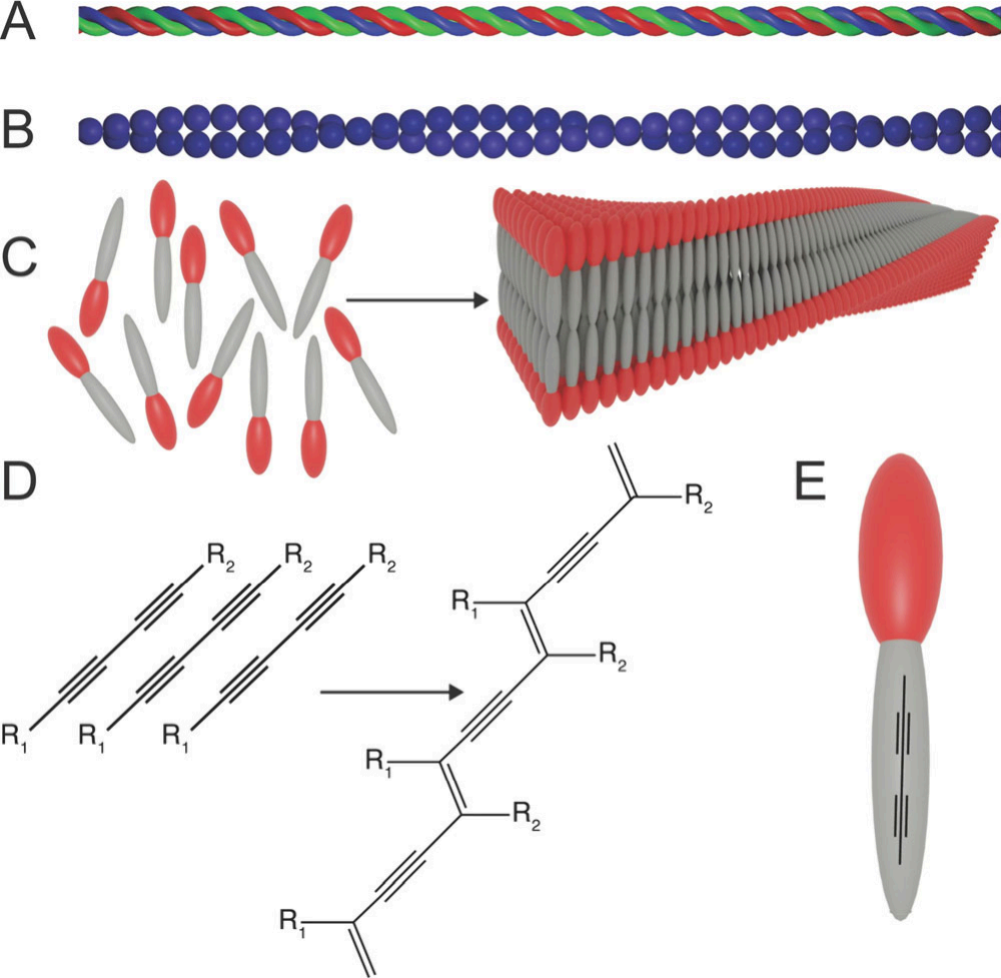
In tissues, filamentous proteins such as (A) collagen or (B) actin give the extracellular matrix and cellular cytoskeleton their mechanical integrity. (C) Self-assembling peptides can be designed such that they form high aspect ratio nanostructures, like matrix proteins. (D) Diacetylene groups can undergo topotactic polymerization to form highly extended polymers, and (E) these moieties can be integrated into the hydrophobic domain of self-assembling peptides.

Self-assembling peptides are versatile platforms that can form nanofibers with dimensions and morphologies comparable to natural fibrous proteins^26^ with persistence lengths exceeding hundreds of microns (Figure 1C).^27–29^ The molecular arrangement within peptide nanostructures can be precisely modulated by altering the peptide sequence, allowing for control over supramolecular organization.^30,31^ Peptides can be functionalized with polymerizable groups to enable the formation of covalent bonds after noncovalent assembly.^32–34^ This is most commonly done through the incorporation of diacetylene groups,^35–40^ which undergo topotactic polymerization (i.e., a polymerization that occurs in a highly ordered lattice in which polymerization induces minimal changes to the lattice structure) to form highly extended covalent polymers down the nanofiber axis (Figure 1D).^35,41^ A key advantage of these peptide-based systems is their spontaneous assembly under aqueous conditions, facilitating integration into diverse polymeric environments and mimicking the multinetwork architectures of natural tissues. This makes self-assembly an ideal motif for the formation of highly extended polymers to stiffen hydrogel matrices.

In this study, we report a series of self-assembling diacetylene peptide amphiphiles (DA-PAs) whose peptide sequences were systematically engineered to precisely control the molecular orientation within self-assembled nanostructures. We discovered that sequences that formed high aspect ratio nanofibers exhibited a significantly enhanced propensity for diacetylene polymerization. DA-PAs were incorporated into poly­(ethylene glycol) (PEG) matrices to form interpenetrating network (IPN) hydrogels, and polymerization of the DA-PAs to form covalent polymer networks led to a 55-fold increase in hydrogel stiffness compared to unpolymerized systems. The molecular interaction between the PEG and DA-PA network was tuned by changing the chemical structure of the cross-linker for the PEG network, and this was found to greatly change both the elastic and viscous components of the system, highlighting the importance of interfacial interactions between the networks. The broad applicability of this system was demonstrated by incorporating DA-PAs into a different hydrogel system, alginate, which also led to substantial improvement in mechanical properties. The insights and design principles established in this work bridge supramolecular assembly and covalent polymer chemistry, paving the way for advanced biomimetic materials capable of recapitulating the complex structural and mechanical functionality of biological tissues.

## Materials and Methods

### Materials

All peptide synthesis reagents were purchased from Chemscene or Ambeed. *N*,*N*-Dimethylformamide (DMF) and dichloromethane (DCM) were obtained from VWR BDH Chemicals, while piperidine and trifluoroacetic acid were supplied by Millipore Sigma. Diethyl ether was acquired from Fisher Scientific and *N*,*N*-diisopropylethylamine (DIPEA), from VWR. 20 kDa 8-arm poly­(ethylene glycol) dibenzocyclooctyne (PEG-DBCO) was purchased from Creative PEGWorks, and N_3_-PEG3-N_3_ was purchased from Lumiprobe Corporation. 10,12-Pentacosadiynoic acid was purchased from BeanTown Chemical. E401 sodium alginate was purchased from Lepicerie. Gelatin from porcine skin was purchased from Sigma-Aldrich.

### Methods

#### Peptide Synthesis

Peptides were synthesized using standard solid phase peptide synthesis protocols using either manual synthesis or an automated peptide synthesizer (CEM Liberty Blue) using standard Fmoc-protected amino acids (Chemscene) on a Rink amide resin (Supra Sciences). All amide couplings were done using *O*-(6-chlorobenzotriazol-1-yl)-*N*,*N*,*N*′,*N*′-tetramethyluronium hexafluorophosphate (HCTU) unless otherwise noted. For each coupling, the amino acid, HCTU, and DIPEA were added in a 4:4:6 ratio to the peptide. During peptide synthesis, a ninhydrin test was performed after every addition to test for the presence of free amines. Upon a positive test, the coupling was replicated until it was negative. After successful coupling, the Fmoc group was removed, and the resin was washed with 20% piperidine in DMF twice for 5 min. A ninhydrin test was performed to check for a positive result. The N-terminus of cross-linking peptides was modified with an azide by coupling an azido acetic acid using HCTU/DIPEA chemistry. 2-Azido acetic acid was synthesized by mixing bromoacetic acid (70.168 g, 505 mmol) and sodium azide (32.504 g, 500 mmol) in water (250 mL) and stirring overnight at RT under ambient conditions. The next day, the solution was acidified to pH ∼ 1 using hydrochloric acid and extracted using diethyl ether (5 × 100 mL). The organic layers were combined and dried in vacuo to afford 2-azidoacetic acid as a colorless liquid. 2-Azidoacetic acid was stored in a −20 °C freezer until it was needed for synthesis.

All peptides were cleaved using 95% trifluoroacetic acid (TFA), 2.5% H_2_O, and 2.5% triisopropylsilane (TIPS). Peptides that contain a tryptophan were cleaved with 2.5% dithiothreitol (DTT). Peptides were typically cleaved for 30 min at room temperature using approximately 2 mL of cleavage solution per mM of peptide. The mass was checked using electrospray ionization, and if protecting groups remained, the peptide was recleaved for 30 min. At the end of the cleavage, the peptides were precipitated in diethyl ether. The precipitated peptides were then centrifuged for 5 min at 4,000 rpm, and the supernatant was discarded. The peptide pellet was washed with diethyl ether and centrifuged, and this was repeated three times. The diacetylene peptide pellets were quickly dried to avoid polymerization and used immediately without further purification.

#### Peptide Purification

N_3_Gly-KGPQGI­WGQKK­(N_3_) was purified using high-performance liquid chromatography (HPLC) on a Phenomenex Gemini 5 μm NX-C18 110 Å LC Column (150 × 21.2 mm). The gradient was run from 95% Mobile Phase A (water containing 0.1% trifluoroacetic acid (TFA)) and 5% Mobile Phase B (acetonitrile containing 0.1% TFA) to 100% Mobile Phase B. Each HPLC run included a 2 min equilibration phase, followed by a 10 min gradient from 95% Mobile Phase A to 100% Mobile Phase B, and a subsequent 2 min equilibration at 100% Mobile Phase B, before returning to the initial conditions. Following purification, the peptide was lyophilized and stored at −20 °C in a freezer.

#### Hydrogel Fabrication

Hydrogels were prepared by sequentially adding 8-arm PEG-DBCO, followed by DA-PAs, and finally the cross-linking peptides to initiate gelation with each component present at the concentration described in Table 1. After gelation, the samples were immersed in mineral oil and exposed to UV irradiation at 365 nm for 45 min using a MAXIMA ML-3500S ultrahigh-intensity UV source (35 W) with the gels positioned approximately 2–3 cm from the lamp to induce polymerization of the diacetylene tails.

**Table 1:** Formulations of Hydrogels Used in These Studies

Condition	Polymer Conc. (% wt/vol)	Cross-Linker Conc.	PA Conc.
V2A2E3/K-PanMMP cross-linked + V2A2E3 PA	10% PEG	40 mM N_3_-KGPQGIWGQK-K(N_3_)	50 mM V2A2E3
E-PanMMP cross-linked + V2A2E3 PA	10% PEG	40 mM N_3_-EGPQGIWGQE-K(N_3_)	50 mM V2A2E3
PEG cross-linked + PA	10% PEG	40 mM N_3_-PEG-N_3_	50 mM V2A2E3
K-PanMMP cross-linked	10% PEG	40 mM N_3_-KGPQGIWGQK-K(N_3_)	0
E-PanMMP cross-linked	10% PEG	40 mM N_3_-EGPQGIWGQE-K(N_3_)	0
PEG cross-linked	10% PEG	40 mM N_3_-PEG3-N_3_	0
V2E2	10% PEG	40 mM N_3_-KGPQGIWGQK-K(N_3_)	50 mM V2E2
V2A2E2	10% PEG	40 mM N_3_-KGPQGIWGQK-K(N_3_)	50 mM V2A2E2
V3A3E3	10% PEG	40 mM N_3_-KGPQGIWGQK-K(N_3_)	50 mM V3A3E3
V2A2E4	10% PEG	40 mM N_3_-KGPQGIWGQK-K(N_3_)	50 mM V2A2E4
V2A2E3-Click	10% PEG	0	10 mM V2A2E3 + 40 mM V2A2E3 click
Alginate	2% Alginate	50 mM CaCl_2_	0
Alginate + V2A2E3 PA	2% Alginate	50 mM CaCl_2_	50 mM V2A2E3
Gelatin	10% Gelatin	0	0
Gelatin + V2A2E3 PA	10% Gelatin	0	50 mM V2A2E3

Hydrogels were prepared at pH 7, with the exception of the pH-dependent studies. In these experiments, all components were adjusted to the target pH prior to gel formation. For instance, for rheology at pH 9, each component solution was prepared at pH 9. After gelation, the samples were immersed in mineral oil. When UV exposure was required, gels were irradiated under these conditions for 45 min.

For swelling experiments, hydrogel samples were prepared as described previously in 24-well plates and polymerized by UV exposure. After polymerization, 1 mL of 1× PBS was added to each well, and the gels were incubated at 37 °C for 24 h to reach swelling equilibrium. After incubation, the gels were removed from PBS, and rheological measurements were performed immediately.

#### Circular Dichroism (CD) Spectroscopy

Each PA sample was diluted to 1 mM in water containing ammonia and adjusted to pH 7. CD spectra were recorded on a JASCO model J-815 spectropolarimeter by using a quartz cell of 0.1 mm optical path length. Continuous scanning mode was used with a scanning speed of 100 nm/min with the sensitivity set to standard mode. High Tension (HT) voltage was recorded for each sample to ensure that the measurement was not saturated. An accumulation of three measurements was used, and a water sample was background-subtracted to obtain the final spectra.

#### Mechanical Testing

All rheological studies used an AR-Ex 2000 or Discovery HR-20 rheometer. Rheology tests were performed on the gels using frequency sweeps at 0.1% strain from 0.1 to 10 Hz and a strain sweep was performed at constant frequency of 1 Hz from 0.01% to 100% using an 8 mm parallel plate. Modulus values were taken from the frequency sweeps at 1 Hz. Each value represents the average of three runs. Stress relaxation tests were conducted for 1200 s at a constant strain of 10%, and the data were normalized to the average of the five time points around 0.025 s. The cyclic strain tests were performed by using a low-strain time sweep (0.1% strain, 1 Hz, 2 min) followed by a high-strain time sweep (100% strain, 1 Hz, 2 min). This cycle was repeated five times.

#### Transmission Electron Microscopy

The self-assembly of PAs was promoted by dissolving the conjugates into deionized water and ammonia to adjust the pH to 7 at a concentration of 10 mM. The solutions were sonicated for 5 min and allowed to sit for 1 h. Stock solutions for TEM were prepared by diluting the solution above to 350 μM. The unstained TEM samples were prepared by adding a 7.5 μL droplet of stock solution onto a 300-mesh ultrathin carbon film on a lacey carbon support film TEM grid. The excess solution was wicked away using Kimwipes after 30 s, and the grid was left to dry before imaging. The TEM images were obtained on a JEOL-2100 TEM instrument operating at 120 kV.

#### Scanning Electron Microscopy (SEM)

For SEM imaging, the hydrogel samples were gradually dehydrated by sequential immersion in ethanol–water solutions of increasing ethanol concentration (from 10:90 to 100:0, v/v). Gradual exchange of water with ethanol is essential to minimize osmotic shock and structural collapse. The dehydrated samples were subsequently subjected to graded replacement with hexamethyldisilazane (HMDS) by immersion in HMDS–ethanol mixtures of increasing HMDS concentration (from 10:90 to 100:0, v/v), followed by drying overnight at room temperature. Stepwise substitution with HMDS ensures gentle drying and reduces the risk of shrinkage and pore collapse. The samples were imaged by a Thermo Fisher Helios 5 PFIB DualBeam SEM.

#### UV–Vis Spectroscopy

UV–vis spectroscopy was performed by using a BioTek Cytation 5 plate reader for time-dependent irradiation experiments at room temperature. Using PA solutions diluted to a concentration of 5 mM, UV–vis absorption was monitored following UV exposure at time intervals of 30 s, 1 min, 2 min, 5 min and subsequently every 5 min for up to 1 h. In the spectra of PA samples, two major absorption peaks were observed near 530 nm (red state) and 630 nm (blue state). Absorbance measurements for tracking DBCO reactivity were performed using a Shimadzu UV–Vis 2600 spectrophotometer equipped with an ISR-2600-Plus integrating sphere attachment. Samples were prepared in a 0.1 mm path length cuvette. To assess reactivity, spectra were collected 10 min after the addition of cross-linkers to 8-arm PEG-DBCO. The concentration of PEG-DBCO used for these measurements was 1 mM.

#### Dynamic Light Scattering (DLS)

All samples were prepared at a concentration of 0.2 g L^–1^ in trifluoroacetic acid (TFA). For polymerized samples, solutions were exposed to UV irradiation for 45 min in an aqueous solution, followed by lyophilization overnight. The resulting powders were redissolved in TFA and sonicated for 2 min in glass tubes prior to analysis. DLS measurements were performed by using an ALV/CGS-3 compact goniometer system. Samples were measured by using a laser wavelength of 632.8 nm at a scattering angle of 173° and a temperature of 25 °C. The hydrodynamic size distributions were obtained by using the built-in regularized fit algorithm provided by the ALV software (ALV-7004).

#### Statistical Analysis

Data were analyzed using a one-way ANOVA followed by a Tukey’s post hoc test in R. The Tukey’s test results can be found in the Supporting Information.

## Results and Discussion

We synthesized a series of peptides with 10,12-pentacosadiynoic acid on their N-termini to generate diacetylene-modified peptide amphiphiles (DA-PAs) (Figure 2A). The peptide sequence within diacetylene-modified peptide amphiphiles was systematically modified to identify self-assembling peptides with a high propensity for polymerization under the hypothesis that DA-PAs with a higher propensity for polymerization will have a greater ability to improve the mechanical properties of DA-PA hydrogels. We first synthesized a series of DA-PAs that have been previously shown to form a diverse set of nanostructures when conjugated with a palmitoyl tail that lacks diacetylene moieties (Figure 2B).^30,42^ We synthesized four different permutations of DA-PAs containing two valines (V) and two glutamic acids (E): (V2E2, VEVE, E2V2, and EVEV). When conjugated with palmitic acid tails, these peptides were shown to form a series of different flat nanostructures.^30^ We also synthesized a DA-PA with two alanines (A) (V2A2E2), which was shown to form thinner, high aspect ratio nanostructures when functionalized with a palmitic acid tail.^42^ Peptide amphiphiles have been extensively studied over the past several decades, which includes understanding how the peptide sequence can be tuned to control supramolecular assembly.^30,43,44^ However, it should be noted that most of the peptides used in these studies are relatively short, having peptide segments with lengths between four and seven amino acids, while the 10,12-pentacosadiynoic acid tail used for every DA-PA in this work is significantly longer than the commonly used palmitic acid tail, containing 25 carbons compared to 16 for palmitic acid. For the series of DA-PAs containing two valines and two glutamic acids, the 10,12-pentacosadiynoic acid tail accounts for approximately 43% of the atomic mass of the molecule.

**Figure 2:**
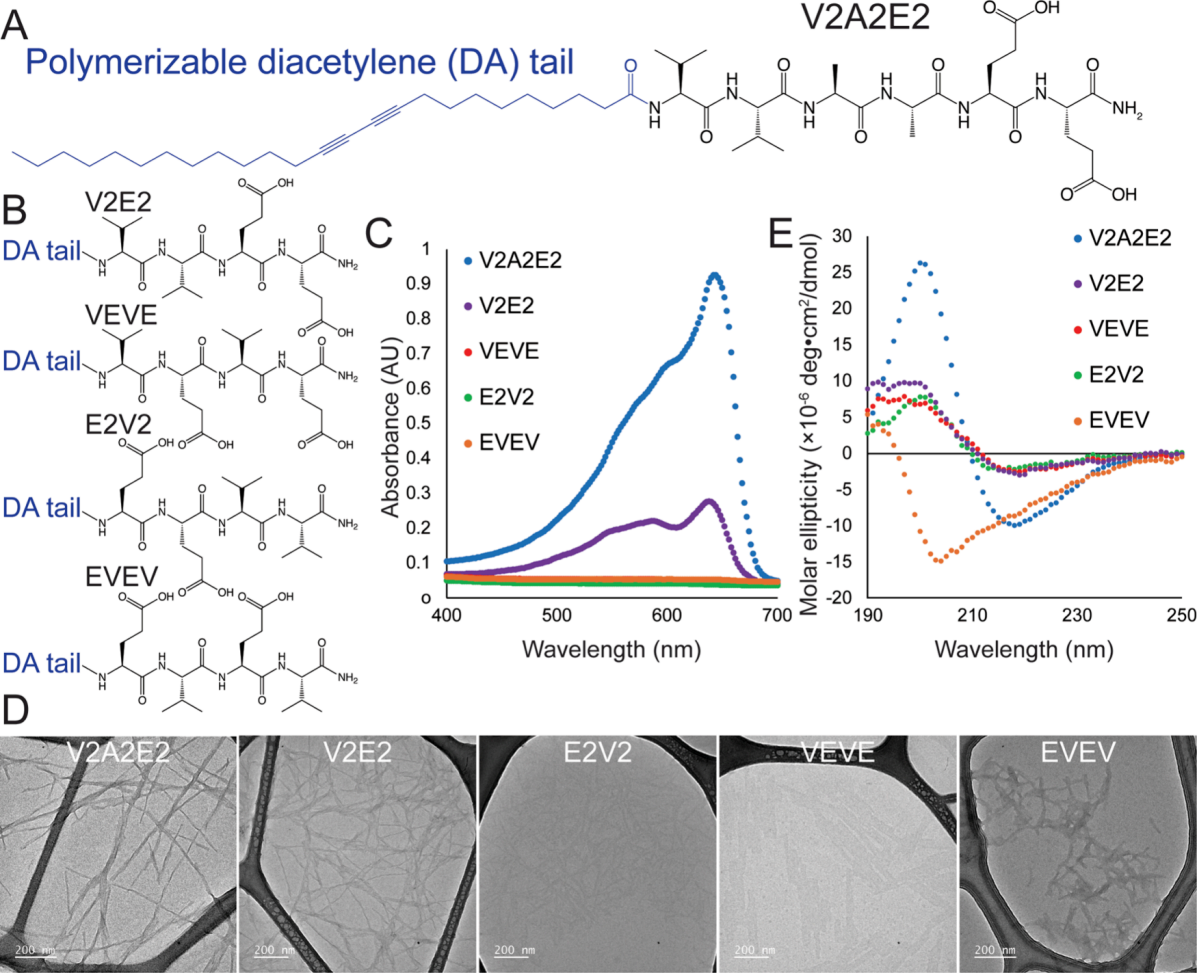
(A) Peptide amphiphiles can be modified with diacetylene functionalities (DA-PAs) to enable covalent polymerization in the supramolecular nanofibers. (B) We designed a series of DA-PAs to identify sequences that maximized diacetylene polymerization. (C) Polymerization of diacetylene groups leads to an increase in absorbance at 640 nm, and we found that the propensity of DA-PAs to undergo polymerization was strongly dependent upon the peptide sequence. (D) Transmission electron microscopy showed that DA-PA sequences that did not readily polymerize, such as E2V2, VEVE, or EVEV, formed flat structures (VEVE) or short fibrils (E2V2, EVEV), while V2E2 and V2A2E2 formed longer fibers. (E) Circular dichroism showed that each of the DA-PAs, except for the EVEV sequence, self-assembled into β-sheets in water.

The diacetylene groups within the 10,12-pentacosadiynoic acid moieties present in the DA-PAs undergo a 1,4-addition topochemical polymerization reaction upon UV irradiation that forms extended polydiacetylene conjugated backbone chains.^45^ This polymerization creates an alternating ene-yne conjugated backbone, giving rise to characteristic absorption bands (Figure 1D).^46^ We found that the ability of the DA-PAs to polymerize was highly dependent upon the peptide sequence (Figures 2C and S1) with three of the five peptides (VEVE, E2V2, and EVEV) failing to undergo noticeable polymerization after 15 min. Both the V2E2 and V2A2E2 peptides polymerized, and V2A2E2 had a greater amount of polymerization, as indicated by the stronger spectroscopic absorbance, and significant differences in peak shape. The absorbance maximum at 640 nm is the “blue phase” of polydiacetylene and is associated with highly ordered, planar, extended polymers.^47^ The absorbance at shorter wavelengths (<600 nm) leads to a reddish or purplish color and indicates shorter effective conjugation lengths or more strained or disordered polymers.^48^ The V2A2E2 peptide has a greater ratio of the 640 nm peak to the shorter wavelength peaks, indicating that the diacetylene polymers that are formed have fewer defects or longer conjugation lengths. We performed transmission electron microscopy (TEM) to understand how differences in peptide sequence influenced the nanostructure of the assembled peptides (Figure 2D). TEM showed that sequences that supported polymerization of diacetylene formed thinner high aspect ratio nanostructures, while those that did not polymerize formed short fibers or aggregates (E2V2, EVEV) or sheet-like structures (VEVE).

Circular dichroism (CD) spectroscopy was performed on the five different DA-PAs to understand how changes in the peptide sequence alter their secondary structure within the nanoscale assemblies. There is a significant amount of spectral diversity of CD signals for β-sheets, which has been shown to be associated with differences in molecular orientation within the β-sheets.^49,50^ We found that four of the DA-PAs had a β-sheet signature, indicated by the positive peak at around 200 nm and the negative peak at 220 nm (Figure 2E). The exception was EVEV, which adopted a mostly random coil conformation, characterized by a strong negative peak near 200 nm. The shifts in these band positions and intensities directly report on variations in interstrand hydrogen-bond geometry and backbone dihedral angles, and the differences between the DA-PAs likely reflects the distinct molecular conformations in each peptide within their supramolecular assemblies.^49^ Notably in our samples, the CD spectra of the V2A2E2 peptide have a significantly diminished positive peak, which is also blue-shifted from the peaks seen in the V2E2, VEVE, and E2V2 peptides (Figure 2E). Comparing the UV–vis data with the CD, we observed that the two peptide sequences that supported polymerization had large negative CD peaks, while also having diminished positive peaks. This indicates that different peptide sequences have altered orientations within the β-sheets, and these subtle differences have a large impact on the ability of diacetylene to polymerize.

We then designed a series of peptides based upon the V2A2E2 DA-PA sequence to better understand how peptide charge and β-sheet length influence the topotactic polymerization of the diacetylenes (Figure 3A). Most self-assembling peptides include charged amino acids to increase solubility in water, and the neutralization or screening of these charges by either changing the pH or adding salts is typically used to induce gelation. The charged amino acids on the peptides also undergo Coulombic repulsion, and varying the amount of charge on the peptides can have a significant influence on the molecular ordering and nanostructures formed by peptides.^51,52^ We synthesized a series of peptides having a V2A2 sequence followed by either two, three, or four glutamic acids and found that the peptide charge has a significant effect on the ability of DA-PAs to polymerize (Figure 3B). The intensity of the blue phase peak at 640 nm for the V2A2E3 peptide was more than twice as great after 15 min of UV exposure than that of either V2A2E2 or V2A2E4.

**Figure 3:**
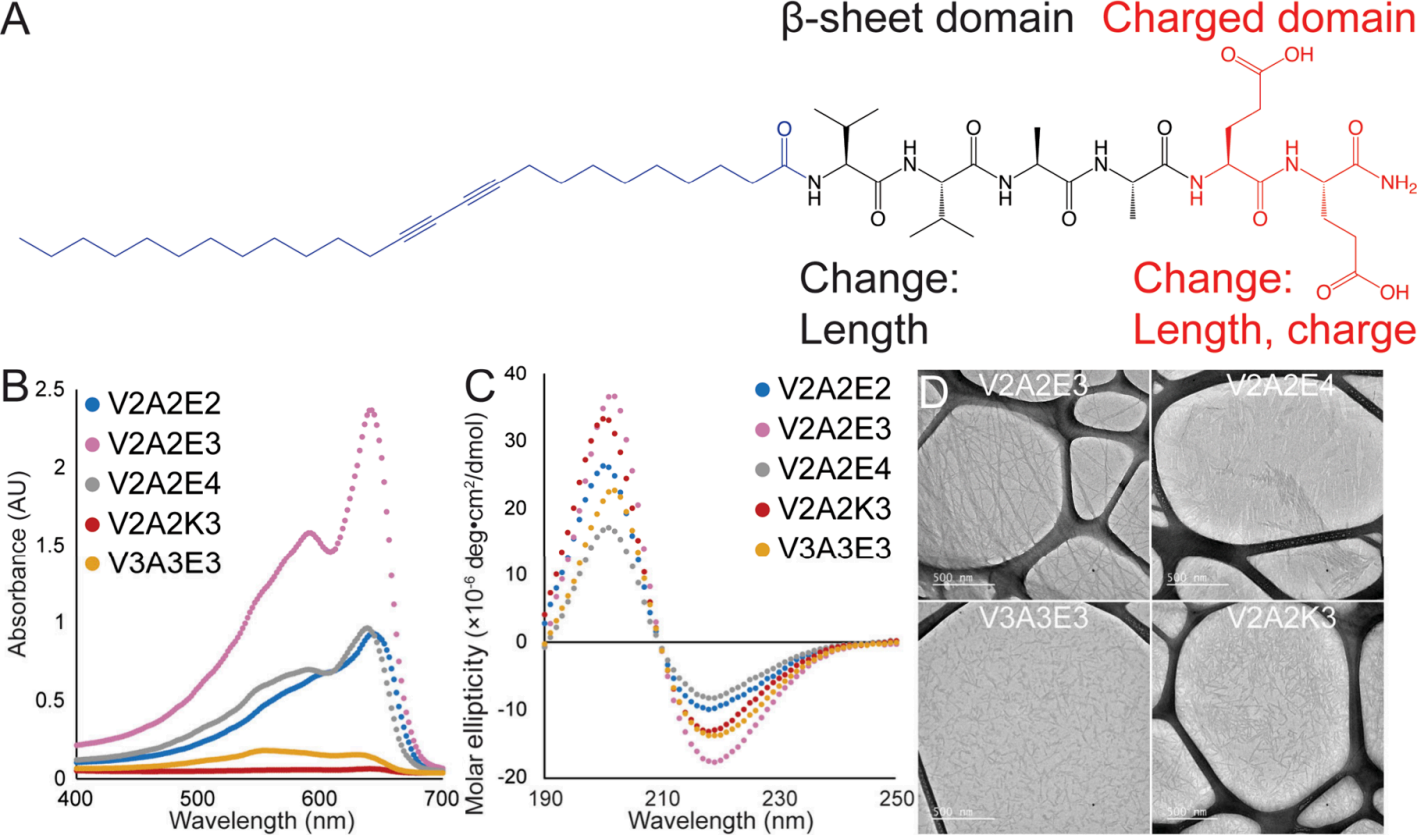
(A) Series of DA-PAs based on the V2A2 sequence. We modified the length of the β-sheet sequence and the number of ionizable amino acids and their charge, testing both negatively charged glutamic acids (E) and positively charged lysines (K). (B) The propensity for the diacetylenes to polymerize was highly dependent on the number of charged amino acids and the length of the β-sheet sequence with the V2A2E3 sequence having substantially faster polymerization kinetics. (C) All five peptides formed β-sheets; however, the ratio of positive and negative peak heights varied, indicating different peptide orientations within the nanostructures. (D) The V2A2E3 PA formed fibrous high aspect ratio nanostructures, while the other peptides formed either sheet-like structures (V2A2E4) or shorter fibrils.

Based upon these results, the V2A2E3 sequence was identified as the most promising sequence, and we further tested the effects of amino acid charge and β-sheet length. The charge of the peptide was changed from −3 to +3 by replacing the glutamic acids (E) with lysines (K), and we found that the V2A2K3 peptide showed negligible polymerization after 15 min of UV exposure (Figure 3B). We then kept the charged region constant with three glutamic acids and lengthened the β-sheet region by synthesizing a V3A3E3 peptide and found that this peptide only had minimal polymerization after 15 min of UV exposure. It also became a reddish color due to the peaks below 600 nm having greater intensity than the peaks above 600 nm. In total, these results indicate that the V2A2E3 peptide has the ideal β-sheet length, number, and types of charged amino acids to form nanostructures that promote the covalent polymerization of polydiacetylene. Other laboratories have synthesized a series of diacetylene-modified self-assembling molecules and found that their propensity for polymerization can be tuned through molecular modifications such as branching through a lysine side chain^35^ or lengthening hydrophilic and hydrophobic segments in bisurea bolaamphiphiles.^22^ Notably, while our DA-PAs failed to polymerize when modified with lysines, other work has shown that both negatively charged DA-VGDDD-COOH and positively charged DA-VGKKK-COOH sequences were capable of UV-polymerization,^39^ highlighting that the influence of specific molecular modifications on diacetylene polymerization is likely highly specific to individual peptide sequences. Dynamic light scattering (DLS) has previously been used to determine the extent of diacetylene polymerization within self-assembled nanostructures.^22^ We attempted to disassemble the covalently polymerized DA-PAs in a range of solvents, including trifluoroacetic acid (TFA), but were unable to measure objects with reasonably sized hydrodynamic radii, even after extensive sonication.

Circular dichroism showed that each of these peptides formed β-sheets in solution (Figure 3C); however, adding extra charged amino acids significantly influenced the shape of the CD spectra. Increasing the number of glutamic acids led to a blue shift in the negative peak, as well as a decrease in intensity, while leading to a redshift in the positive peak and an increase in intensity. Switching the charge of the amino acids from negative (V2A2E3) to positive (V2A2K3) only modestly changed the CD spectra, while extending the length of the β-sheet region (V3A3E3) has a more dramatic effect, greatly increasing the negative peak and redshifting and decreasing the positive peak. We performed CD on V2A2E3 after UV exposure to understand the influence that the topotactic polymerization of the diacetylene groups has on the secondary structure of the peptides (Figure S2A). The CD spectra undergo only minor changes upon polymerization with the negative peak at 218 nm increasing slightly in intensity. We also performed Raman spectroscopy and FTIR on the V2A2E3 DA-PA before and after UV polymerization and saw only minor changes in the spectra (Figure S2B,C). The V2A2E3 DA-PA formed high aspect ratio nanofibers in TEM (Figure 3D), while the V3A3E3 and V2A2K3 formed short fibers and the V2A2E4 formed sheet-like structures. TEM was performed on the V2A2E3 peptide after UV exposure, and it was found that the dimensions of the nanofibers were similar before and after diacetylene polymerization (Figure S2D). The CD results indicate that most peptides are contained within β-sheets, and the peptide sequences are mostly similar across systems. Within this context, it is notable that adding a single amino acid, such as changing the sequence from V2A2E3 to V2A2E4, can significantly change the nanostructure (fibers to sheets) and greatly reduce the propensity for polymerization.

### Effect of DA-PA Incorporation into Hydrogels on Mechanical Properties

Previous studies have shown that incorporating supramolecular nanofibers within covalent polymer matrices to form interpenetrating polymer networks can modulate and improve their mechanical properties.^53–56^ To understand how covalent polymerization of the DA-PAs influenced the mechanical properties of the hydrogels, we performed rheometry on each of the DA-PAs that underwent polymerization. We generated a covalent polymer network by cross-linking 20 kDa 8-arm poly­(ethylene glycol) (PEG) functionalized with azide-reactive dibenzocyclooctyne (DBCO) groups with a positively charged N_3_-KGPQGIWGQK-K­(N_3_) (“K-PanMMP”) peptide functionalized with an azide on both termini (Figure 4A). Multiarm PEG hydrogels are often utilized as cell culture matrices, and the K-PanMMP peptide was selected because of its widespread use within biomaterials due to the ability of the PanMMP sequence to be degraded by cell-secreted enzymes such that cells can spread and migrate within these gels.^57^ Since DA-PAs readily dissolve in water, they can be easily mixed with the azide-functionalized cross-linking peptide to generate gels in which the PA nanofibers form an interpenetrating network with the covalent PEG network (Figure 4B).

**Figure 4:**
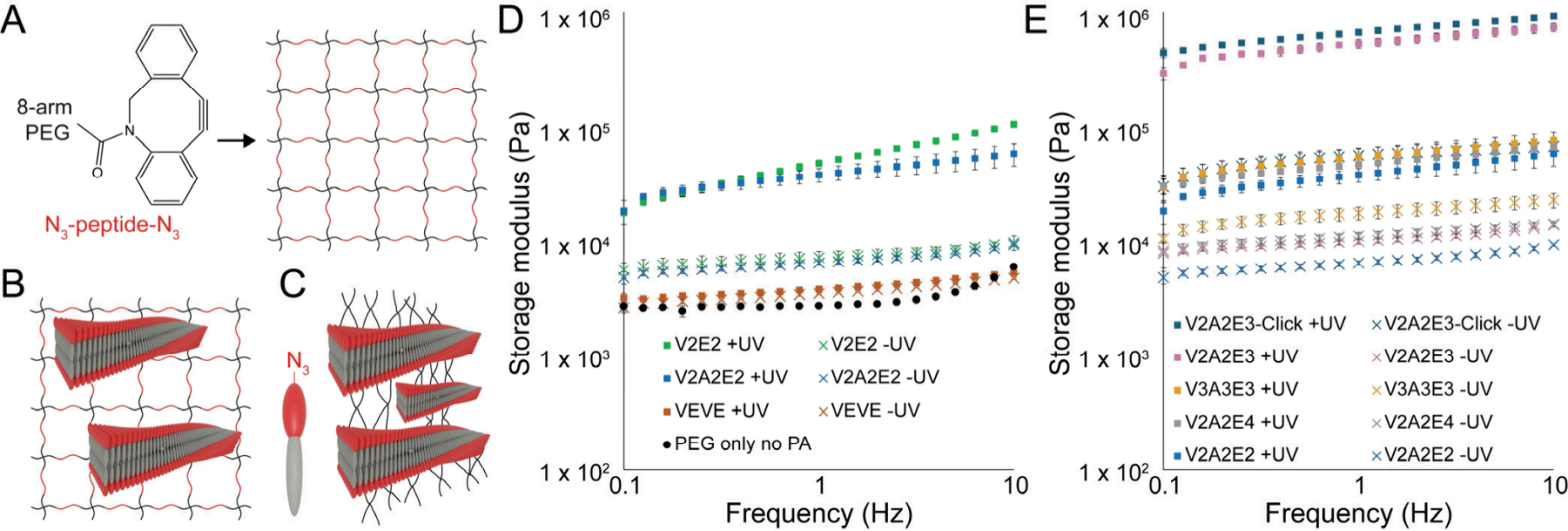
(A) Covalent hydrogel networks were made by cross-linking an 8-arm PEG macromer functionalized with a DBCO strained alkyne with an N_3_-KGPQGI­WGQK-K­(N_3_) peptide functionalized with azides on both termini. (B) Self-assembling peptide nanofibers can be incorporated into the covalent networks to form interpenetrating network hydrogels. (C) Functionalizing the self-assembling peptides with azides and mixing them with 8-arm PEG DBCO forms hydrogels in which self-assembled nanofibers are covalently cross-linked by PEG chains in V3A3E3-Click hydrogels. (D) DA-PAs significantly increase the stiffness of PEG hydrogels. Comparing peptides from the initial screen, sequences that underwent polymerization (V2E2 and V2A2E2) had a significant increase in stiffness upon UV polymerization (+UV), while a sequence that did not polymerize (VEVE) had only a minimal increase. (E) The V2A2E3 peptide, which had the greatest propensity for polymerization, increased the stiffness of PEG hydrogels by over an order of magnitude after polymerization compared with other sequences. Error bars represent ± standard deviation, *N* = 3. Statistical analyses can be found in the Supporting Information.

Peptide amphiphiles can be modified with azides through the use of non-natural amino acids such as azidolysine, which can be used to form hydrogels in which polymers are cross-linked PA nanostructures and not bifunctional cross-linking peptides (V2A2E3-Click) (Figure 4C).^58^ Each of the hydrogels has 40 mM DBCO groups and 40 mM total azide concentration to ensure 1:1 stoichiometry. Since the DA-PAs are present in the hydrogel at 50 mM, for the V2A2E3-Click system, we combine 40 mM V2A2E3-N3 and 10 mM V2A2E3 without the azide functionality. All of the gels studied here contained 10% PEG by weight with a 50 mM concentration of DA-PAs. It should be noted that, while negatively charged peptide amphiphiles are often gelled with cations such as calcium chloride or physiological buffers, the DA-PAs in this work are in pure water unless otherwise noted. The DA-PAs form liquids that can be easily pipetted prior to UV treatment, and while treatment with UV does not induce gelation, their viscosity increases sufficiently so that pipetting the liquids becomes difficult. To identify the length of UV irradiation it takes to maximize the mechanical properties of hydrogels, we took the DA-PA with the slowest polymerization kinetics, V3A3E3, and performed rheometry at time points ranging from 45 to 120 min of UV exposure (Figure S4). These studies showed minimal differences in mechanical properties between the time points, and all subsequent rheometry on UV-polymerized gels was done with 45 min of irradiation. To validate the presence of high aspect ratio nanostructures within the UV-polymerized gels, we performed scanning electron microscopy on hydrogels without DA-PAs and with the V2A2E3 DA-PA both before and after UV-polymerization (Figure S4). We found that PEG hydrogels and hydrogels with unpolymerized V2A2E3 DA-PA formed gels without noticeable nanoscale features (Figure S3A,B), while UV-irradiated V2A2E3 DA-PA gels had high aspect ratio nanostructures with diameters of 30–60 nm, validating the presence of nanofibers within the gels.

We first tested two peptides from the initial set of sequences which underwent UV polymerization, V2E2 and V2A2E2, and one which did not, VEVE (Figure 4D). Our data show that polymerization is required for an improvement in hydrogel mechanical properties after UV exposure, as both V2E2 and V2A2E2 had greater than 6-fold increases in storage modulus (*p* < 0.001), while the increase in storage modulus for VEVE only increased by 10%, which is not statistically significant. We found that the incorporation of DA-PAs significantly increased the storage modulus of hydrogels compared to PEG gels, which did not have supramolecular networks, even before UV polymerization (Figure 4D,E, Table 2) (*p* < 0.001). Prior to UV polymerization, there was a general trend in which DA-PAs with longer peptide sequences formed stiffer gels, with the V3A3E3 peptide forming the stiffest gel, followed by V2A2E4. Since the extent of hydrogen bonding between peptides is dependent upon the length of the peptide sequence, it is likely that longer peptide sequences feature greater intermolecular bonding and are more effective at resisting deformation. Interestingly, the storage modulus of the hydrogels more than tripled when DA-PA nanofibers were covalently cross-linked by PEG (Figure 4D, V2A2E3-Click in Table 2), despite lacking covalent cross-links in the PEG network. The storage modulus of hydrogels depends upon the cross-linking functionality, and moving from cross-linking 8-arm PEG with bifunctional cross-links, such as peptides flanked with azides, to azide-functionalized nanofibers forms networks in which the supramolecular polymers act as central cross-linking foci with very high functionality. Previous work in peptide-conjugated polymers has also shown that stiff hydrogels can be formed via noncovalent interactions.^58–60^

**Table 2:** Mechanical Properties of the DA-PA Hydrogels^a^

	Without UV		With UV	
Condition	Storage Modulus (kPa)	Loss Modulus (kPa)	Storage Modulus (kPa)	Loss Modulus (kPa)
K-PanMMP (no PA)	2.78 ± 0.08	0.11 ± 0.05		
VEVE	3.57 ± 0.08	0.68 ± 0.03	3.94 ± 0.12	0.74 ± 0.02
V2E2	7.48 ± 0.67	0.83 ± 0.11	50.9 ± 3.35	30.8 ± 0.86
V2A2E2	6.68 ± 0.32	1.07 ± 0.03	40.2 ± 5.57	11.6 ± 4.08
V2A2E3	10.4 ± 0.55	1.43 ± 0.19	579 ± 50.6	133 ± 13.9
V2A2E4	11.6 ± 0.85	1.68 ± 0.10	49.7 ± 2.10	10.9 ± 0.53
V3A3E3	18.4 ± 2.37	2.87 ± 0.42	57.7 ± 10.9	13.2 ± 2.28
V2A2E3 Click	59.7 ± 9.58	7.49 ± 1.22	728 ± 27.8	165 ± 6.28

a All hydrogels are 10% 8-arm 20 kDa PEG with a 50 mM total PA concentration. All values are taken from the frequency sweep at 1 Hz.

Polymerization of the DA-PA nanofibers led to a dramatic increase in hydrogel stiffness; however, the magnitude of increase in mechanical properties was dependent upon the peptide sequence (Figure 4D,E, Table 2). For most of the DA-PAs, the storage modulus of the hydrogel increased from 6–18 kPa to 40–57 kPa, typically increasing 3× to 6× upon polymerization of the diacetylene moiety. Hydrogels containing the V2A2E3 DA-PA were a significant exception to this trend, and these hydrogels had a 55-fold increase in storage modulus upon DA-PA polymerization. These matrices were more than 10× stiffer than those containing other peptides, having a storage modulus of 578.5 ± 50.6 kPa after UV polymerization, compared to 10.4 ± 0.55 kPa before polymerization. Covalently coupling the V2A2E3 DA-PA nanofibers to PEG via an azide modification further increased the hydrogel stiffness after polymerization, resulting in a material with a stiffness of 728 ± 27.8 kPa (Table 2). The stiffness of all hydrogels modestly increased at higher oscillation frequencies (Figure 4D) and generally exhibited a trend in which the mechanical properties of stiffer hydrogels degraded at lower strains than those of softer hydrogels (Figure S5). To further validate the importance of a polymerizable diacetylene moiety in the increase in mechanical properties of DA-PA hydrogels, we functionalized the N-terminus of the V2A2E3 peptide with a 25 carbon pentacosanoic acid, which has the same number of carbons as the diacetylene tail but contains no double or triple bonds. Rheology performed on these hydrogels indicated that they have mechanical properties that are roughly equivalent to the unpolymerized DA-PAs, while being dramatically lower than those of the UV polymerized DA-PA gels (Figure S7).

It is notable that the benefits of the V2A2E3 sequence over other peptides became apparent only after covalent polymerization of the diacetylene unit. There are significant differences in the morphology of the nanostructures formed by different peptide sequences (Figures 2E and 3C), but the rheological results show that the viscoelastic properties of PEG–PA gels before polymerization were mostly influenced by the length of the peptide sequence and not on the nanostructure (Table 2). After polymerization, both the storage and loss moduli for the V2A2E3 peptide are 10× greater than those of V2A2E4 and V3A3E3, despite being a shorter peptide sequence (*p* < 0.001). The V2E2, V2A2E2, and V2A2E3 PAs formed a thin high aspect ratio nanostructure, and V2A2E3 had the highest propensity for diacetylene polymerization (Figure 3B). We quantified the propensity for DA-PAs to undergo UV polymerization by integrating the area under the curve in UV–vis spectroscopy from 400 to 700 nm. This was compared to the improvement effect that UV polymerization had on mechanical properties, as measured by the fold increase in modulus, and we found that there was a general positive trend in which DA-PAs that polymerized the fastest had the greatest increases in mechanical properties (Figure S7).

The mechanical properties of hydrogels are sensitive to factors aside from the molecular structure, including the pH of the water and time-dependent changes in hydrogel volume due to swelling. Most self-assembling peptides, including the DA-PAs studied here, contain charged amino acids, and changes in ionization can modulate the orientation of molecules within the assembled nanostructures. In order to better understand the effect of pH on diacetylene polymerization, we irradiated the V2A2E3 DA-PA at both acidic pH (5) and basic pH (9) in addition to neutral pH (7) used in the previous studies. We found that the ability of the diacetylenes to polymerize was highly dependent upon pH: polymerization was maximized at pH 7, significantly reduced at pH 5, and only minimal at pH 9 (Figure S8). Decreasing the pH of negatively charged peptide amphiphile hydrogels can often lead to gelation, and the decrease in polymerization was unexpected. However, previous work has shown that the rate of covalent bond formation within self-assembled peptides can be lower within more assembled systems.^61^ This highlights how the reaction efficiency can be tied to specific molecular orientations and is not directly proportional to molecular order. The reduction in polymerization decreased hydrogel mechanical properties with pH 5 gels having a storage modulus of 307 ± 21.8 kPa and pH 9 gels having a storage modulus of 56.2 ± 4.27 kPa, compared to 578.5 ± 50.6 kPa for gels at pH 7 (Figure S9). The influence of swelling on hydrogel behavior was quantified by incubating V2A2E3 DA-PA gels polymerized at pH 7 in phosphate buffered saline (PBS) at 37 °C for 24 h. We found that swelling in PBS decreased the storage modulus of the hydrogels from 578.5 ± 50.6 kPa to 133 ± 25.3 kPa (Figure S10).

The storage modulus (*G*′) of a hydrogel quantifies the elastic energy-storing component of the mechanical properties, while the loss modulus (*G*″) represents the viscous energy-dissipating component. Their ratio, known as tan δ (*G*″/*G*′), describes the relative contribution of viscous to elastic behavior and provides a direct measure of energy dissipation with higher values indicating more pronounced viscous losses during deformation. Covalent hydrogels, such as the PEG network without DA-PAs, are typically purely elastic, since they do not contain bonds that are capable of relaxing stresses. While the PEG only control gel is soft, with a storage modulus of 2.8 kPa, the mechanical properties remain constant to 100% strain (Figure S5A). The ability of the aligned hydrogen bonding down the long axis of peptide amphiphile nanofibers to break and reform is a mechanism of viscous dissipation of energy. Diacetylene polymerization was expected to both increase elasticity by forming new fully extended polymers while also covalently stabilizing the β-sheets, which should reduce their propensity for hydrogen bonds to break. However, while the storage modulus of V2A2E3 hydrogels increased by a factor of 55 after covalent polymerization, the loss modulus increased 93-fold, going from 1.4 ± 0.15 kPa to 133 ± 13.9 kPa (Figure S5). As a result, the tan δ increased for every DA-PA hydrogel after UV polymerization, going from an average value of 0.14 ± 0.002 to 0.30 ± 0.15 (*p* < 0.05). These results indicate that, while the hydrogels are much stiffer, the viscous component of the gels more than doubled.

The formation of highly extended polymers running down the long axis of V2A2E3 nanofibers is likely to increase the stiffness of the V2A2E3 hydrogels, but the even more dramatic increase in loss modulus suggests that there are also new mechanisms for energy dissipation. While the individual DA-PAs form a range of nanostructures, a common feature among them is that their width is generally greater than 15 nm (Figure 3D), which is on the upper end of the mesh size for multiarm PEG hydrogels.^62^ This likely requires the DA-PA nanostructures to be in very close contact with the covalent PEG network. Prior to polymerization, PAs improve the mechanical properties of the hydrogels by requiring that intermolecular hydrogen bonds be broken for gel deformation. To the extent that polymerization covalently stabilizes high aspect-ratio nanostructures, this will prevent nanofiber fracture upon applying shear deformations. Given that the PA nanofibers comprise highly extended polymers while PEG is highly flexible, we hypothesize that upon deformation the covalent PA nanostructures slide through the PEG network. This could create interfacial friction between the two polymer networks, generating a new mechanism for viscous dissipation that does not exist prior to covalently polymerizing the DA-PAs.

The inclusion of rigid high aspect ratio structures in a bulk matrix to improve mechanical properties has been widely studied in the field of fiber-reinforced composites,^63^ where it is well understood that the mechanical properties of the material depend not only on the properties of each phase but also on the interface between the phases. This is also seen in biological systems, where the interactions between collagen and the local matrix is important for tissue mechanics,^64^ and inside of cells, where actin-binding proteins like filamin have roles in force transmission.^16^ In our system, the interfacial interactions between the DA-PA and PEG networks will be heavily dependent upon the extent and types of bonding between the two polymer phases. Since the chemical structure of the DA-PA was already optimized for diacetylene polymerization, we then modified the properties of the PEG network to modulate the internetwork interactions. The peptide used to cross-link the PEG macromers has the sequence N_3_-KGPQGI­WGQK-K­(N_3_) (K-PanMMP) in which two positively charged lysine residues (K) can have significant electrostatic interactions with the glutamic acid residues (E) present on the surface of the V2A2E3 DA-PA nanostructures. To better understand the role that electrostatic interactions have on the viscoelastic properties of the hydrogel, we synthesized versions of the V2A2E3 hydrogel in which we changed the cross-linker of the 8-arm PEG macromer to either an uncharged PEG3 chain (PEG cross-linked) or a negatively charged version of the peptide in which the lysines are replaced by glutamic acids (N_3_-EGPQGI­WGQE-K­(N_3_), E-PanMMP cross-linked). To ensure that any changes in mechanical properties were not due to differences in reactivity between the DBCO on the PEG and the azides on the peptide or PEG, we performed UV–vis spectroscopy to validate that all cross-linkers had nearly complete reactivity with the PEG-DBCO after 10 min (Figure S11).

Prior to polymerization, the PEG cross-linked and K-PanMMP cross-linked hydrogels had equivalent stiffnesses (12.9 ± 1.7 kPa and 10.4 ± 0.6 kPa, respectively), while the E-PanMMP cross-linked gels had stiffnesses that were significantly higher than both, with a storage modulus of 24.8 ± 1.5 kPa (*p* < 0.001) (Figure 5A). Although changing the cross-linker to either the neutral PEG or an anionic E-PanMMP peptide slightly increased the diacetylene polymerization rate compared to the cationic K-PanMMP (Figure S12), the K-PanMMP cross-linked hydrogel was mechanically superior after polymerization (Figure 5). The storage modulus of the K-PanMMP hydrogels (578 ± 51 kPa) was significantly higher than that of the E-PanMMP (119 ± 2 kPa) or the PEG cross-linked gels (58.3 ± 14 kPa) (*p* < 0.001). The K-PanMMP and PEG cross-linked hydrogels have similar viscoelastic properties before UV polymerization, and the PEG cross-linked hydrogels had better polymerization kinetics; yet, the storage modulus of the K-PanMMP hydrogel after polymerization was 9.9× higher than that of the PEG cross-linked hydrogel. Interestingly, polymerizing the PEG cross-linked hydrogel increases the storage modulus by a factor of 4.5, but switching the cross-linker from the polymerized gels from PEG cross-linking to K-PanMMP cross-linking had an even larger effect than polymerization, increasing the stiffness 9.9×.

**Figure 5:**
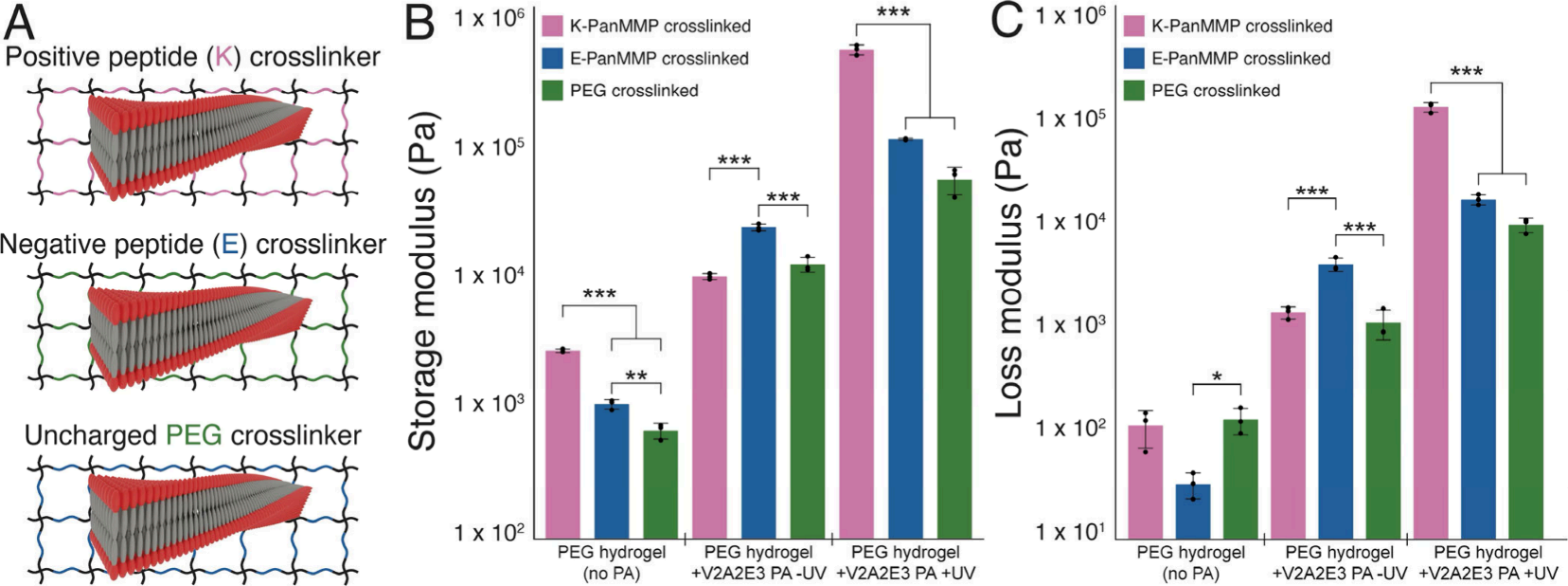
Mechanical properties of hydrogels are highly dependent on interactions between the networks. (A) The negatively charged V2A2E3 DA-PA was incorporated into 8-arm PEG hydrogels with different cross-linkers. (B) Polymerizing the V2A2E3 PA led to a 4.5–4.7 fold increase in storage modulus for the PEG hydrogels with uncharged cross-linkers (PEG) or negatively charged cross-linkers (E-PanMMP) but a 55-fold increase for positively charged cross-linkers (K-PanMMP). (C) Polymerizing the V2A2E3 PA led to a 4-fold increase in loss modulus for the E-PanMMP gels, an 8.7-fold increase in loss modulus for the PEG cross-linked gels, and a 93-fold increase in loss modulus for the K-PanMMP gels. Error bars represent ± standard deviation; * indicates *p* < 0.05, ** indicates *p* < 0.01, and *** indicates *p* < 0.001; *N* = 3.

The effects of the cross-linker chemistry on the loss modulus of UV-polymerized gels was even more pronounced. In unpolymerized gels, the K-PanMMP cross-linked and PEG cross-linked gels had similar loss moduli, 1.4 ± 0.18 kPa and 1.1 ± 0.36 kPa, respectively, while E-PanMMP had a higher modulus value of 4.2 ± 0.61 kPa. Polymerization led to a 4.2× increase in loss modulus for the E-PanMMP hydrogel and an 8.7× increase for the PEG cross-linked gel but a 93× increase in loss modulus for the K-PanMMP gel. Despite the K-PanMMP gel being significantly stiffer, the tan δ for the K-PanMMP cross-linked gel after polymerization (0.23) was significantly higher than that for either the E-PanMMP cross-linker (0.15) or the PEG cross-linker (0.17) (*p* < 0.05) (Figure S13).

Peptide cross-linkers differ from the PEG cross-linker in that they can interact with the PA network by forming both hydrogen and ionic bonds, in addition to being less flexible than PEG. The polymerized E-PanMMP hydrogel is 2× as stiff as the PEG cross-linked gels, indicating the importance of noncovalent interactions between the cross-linking peptides and supramolecular polymers. However, hydrogels with the K-PanMMP cross-linker were 4.9× as stiff as the E-PanMMP cross-linker. Since these peptides are identical, aside from switching two lysines to glutamic acids, this demonstrates the specific importance of ionic bonding as a mechanism to increase intermolecular interactions between the two polymer networks. Such reversible interactions between charged domains may act as sacrificial bonds that dissipate energy without compromising network integrity, mimicking aspects of energy-dissipating mechanisms seen in biological tissues.^65^

To further understand how dynamic bonding influences viscoelastic properties, we performed cyclic recovery studies and stress relaxation experiments on DA-PA hydrogels. The ability of viscoelastic materials to recover after being subjected to high strains can be quantified by subjecting the gels to alternating 2 min intervals of high strains (100%), under which these hydrogels lose most of their storage modulus (Figures S5B and S14), or a low strain (0.1%) that falls within the linear viscoelastic regime of the hydrogels.^66^ We found that PEG hydrogels without any peptide amphiphiles had storage and loss moduli, which were largely equivalent between periods of high and low strain (Figure S14A). Self-assembled systems, on the other hand, typically show shear thinning behavior,^67^ and in our materials, hydrogels with DA-PAs had reduced storage moduli upon the application of 100% strains (Figure S14B–F). All DA-PA hydrogels recovered most of their stiffness during cyclic periods of high strain. All hydrogels with peptide-cross-linked PEG recovered greater than 90% of the original stiffness, even after multiple periods of 100% strain, while hydrogels with PEG cross-links had less than 65% recovery. The decrease in storage moduli for hydrogels with polymerized V2A2E3 was much greater than that for hydrogels with either unpolymerized V2A2E3 or polymerized V3A3E3. Hydrogels with polymerized V2A2E3 had much greater decreases in stiffness during periods of 100% strain and also had a decrease in loss modulus, whereas both unpolymerized V2A2E3 hydrogels and polymerized V3A3E3 had an increase in loss modulus during periods of high strain. These results show that polymerized V2A2E3 DA-PAs led to unique matrix interactions within the cross-linked hydrogel, compared to other unpolymerized V2A2E3 gels or other peptide sequences.

We then performed stress relaxation experiments in which a 10% strain was placed upon the gel and the decay in stresses was quantified. Relaxation studies can be used to understand the time scale of dynamic interactions within self-assembled hydrogels,^68^ and each of the DA-PA hydrogels underwent significant stress relaxation after 1 s, unlike PEG gels without PAs (Figure S15). However, it should be noted that, unlike other peptide sequences, the UV-polymerized V2A2E3 hydrogels showed a substantial reduction in mechanical properties upon a 10% strain (Figure S5B), and this is likely to limit the ability of stress relaxation studies to probe the bond dynamics within these materials. We did find that polymerized V2A2E3 relaxed a higher fraction of initial stresses than other systems, largely due to their higher initial stiffness, as the V2A2E3 K-PanMMP hydrogels had the most stress remaining, in absolute terms, after 1,200 s compared to any other hydrogel system.

A key benefit of using self-assembling peptides to template the formation of covalent networks is that they can be readily dissolved in water and incorporated into other hydrogel systems. Alginate is a natural polysaccharide, and gelatin is derived from collagen; both are widely used as biomaterials in either unmodified or modified forms.^69,70^ Alginate was selected because it is a noncovalent gel, and gelation is induced through the addition of divalent cations, in contrast to the gelation of multiarm PEG macromers using covalent cross-links. A 2% alginate hydrogel with 50 mM CaCl_2_ had a storage modulus of 7.0 ± 1.0 kPa. Adding the V2A2E3 PA to alginate increased it to 18 ± 1.2 kPa, and covalently polymerizing the diacetylene increased it further to 136 ± 8.8 kPa (Figures 6 and S16). Since alginate hydrogels form via noncovalent interactions, they have significant viscous dissipation, and the tan δ values of alginate gels and alginate gels with DA-PAs before and after polymerization were between 0.24 and 0.26. It should be noted that the alginate was gelled with the addition of calcium ions, which will also gel negatively charged peptide amphiphiles like V2A2E3. Adding UV-polymerized DA-PAs to alginate led to an almost 20-fold increase in the storage and loss moduli. Similarly, polymerizing the V2A2E3 DA-PA increased the storage and loss moduli 30× and 50×, respectively, compared to the unpolymerized DA-PA gels, and 217× and 1476× compared to gelatin hydrogels without DA-PAs (Figure 6). These results highlight the ability of this system to improve the mechanical properties of a range of hydrogel systems.

**Figure 6:**
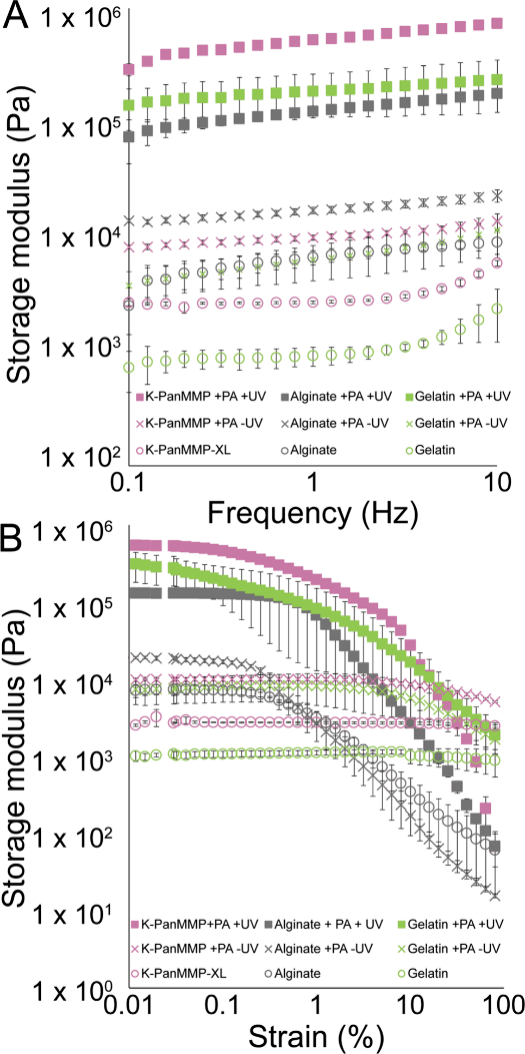
Diacetylene-modified V2A2E3 peptide amphiphiles can be incorporated into other hydrogels to improve mechanical properties, such as alginate and gelatin. The PEG gel was cross-linked with the K-PanMMP peptide. (A) A 2% alginate hydrogel had a storage modulus of 7 kPa. Adding V2A2E3 PA increased the modulus to 18 kPa, and polymerizing the PA increased it further to 136 kPa. 10% Gelatin hydrogels had a storage modulus below 1 kPa; the addition of V2A2E3 PA increased it to 6.5 kPa, and polymerizing the DA-PA increased it further to 203 kPa. (B) Polymerizing the diacetylene-modified PA not only increases the storage modulus for alginate and gelatin gels but also increases the ability of the alginate hydrogels to maintain mechanical integrity as a function of strain. Error bars represent ± standard deviation, *N* = 3. Statistical analyses can be found in the Supporting Information.

## Conclusions

In this work, we designed a series of self-assembling peptide amphiphiles that contain a polymerizable diacetylene moiety and systematically tuned the structure to identify a sequence, V2A2E3, which maximized the polymerization kinetics. When incorporated into covalent peptide-cross-linked PEG hydrogels, these DA-PAs increased the storage modulus 55-fold and the loss modulus 93-fold compared to gels with unpolymerized DA-PAs and 200-fold and 1,000-fold over gels without DA-PAs. Forming PEG hydrogels with cross-linkers that are either positively charged peptides, negatively charged peptides, or neutral PEG linkers dramatically modified the mechanical properties of the hydrogel, highlighting the importance of interactions between the self-assembled and covalent polymer networks. Adding DA-PAs to other hydrogel systems, such as alginate, also led to dramatic improvements in their mechanical properties. While the use of UV light can introduce biocompatibility issues for clinical applications, the formation of highly extended polymers into IPN hydrogels introduces new mechanisms of stiffening hydrogels and energy dissipation that can be applied across most hydrogel systems and to a range of biomedical targets.

## Supplementary Material



## Notes

Quantification of polymerization rates, additional rheology, peptide characterization, and statistical analysis (PDF)

## Abbreviations

PA: peptide amphiphile
PEG: poly­(ethylene glycol)
DA-PA: diacetylene peptide amphiphile
CD: circular dichroism
TEM: transmission electron microscopy
