# Supplementary material for: Programming Hydrogel Mechanics via Sequence-Controlled Polymerization Using Peptide Self-Assembly
Source: J Am Chem Soc. 2026 Jan 29;148(5):4938–50. doi: 10.1021/jacs.5c12182 (PMC12903843; doi:10.1021/jacs.5c12182)
Supplement: Supplementary file 1 [file ja5c12182_si_001.pdf]

Programming Hydrogel Mechanics via Sequence-Controlled Polymerization using Peptide Self-Assembly

Abolfazl S. Moghaddam,[1] Maahi Zaman,[1] Sz-Chian Liou,[2] E. Thomas Pashuck[1*]
[1]Department of Bioengineering, Lehigh University, Bethlehem, PA 18015, USA
[2]Institute for Functional Materials and Devices, Lehigh University, Bethlehem, PA 18015, USA

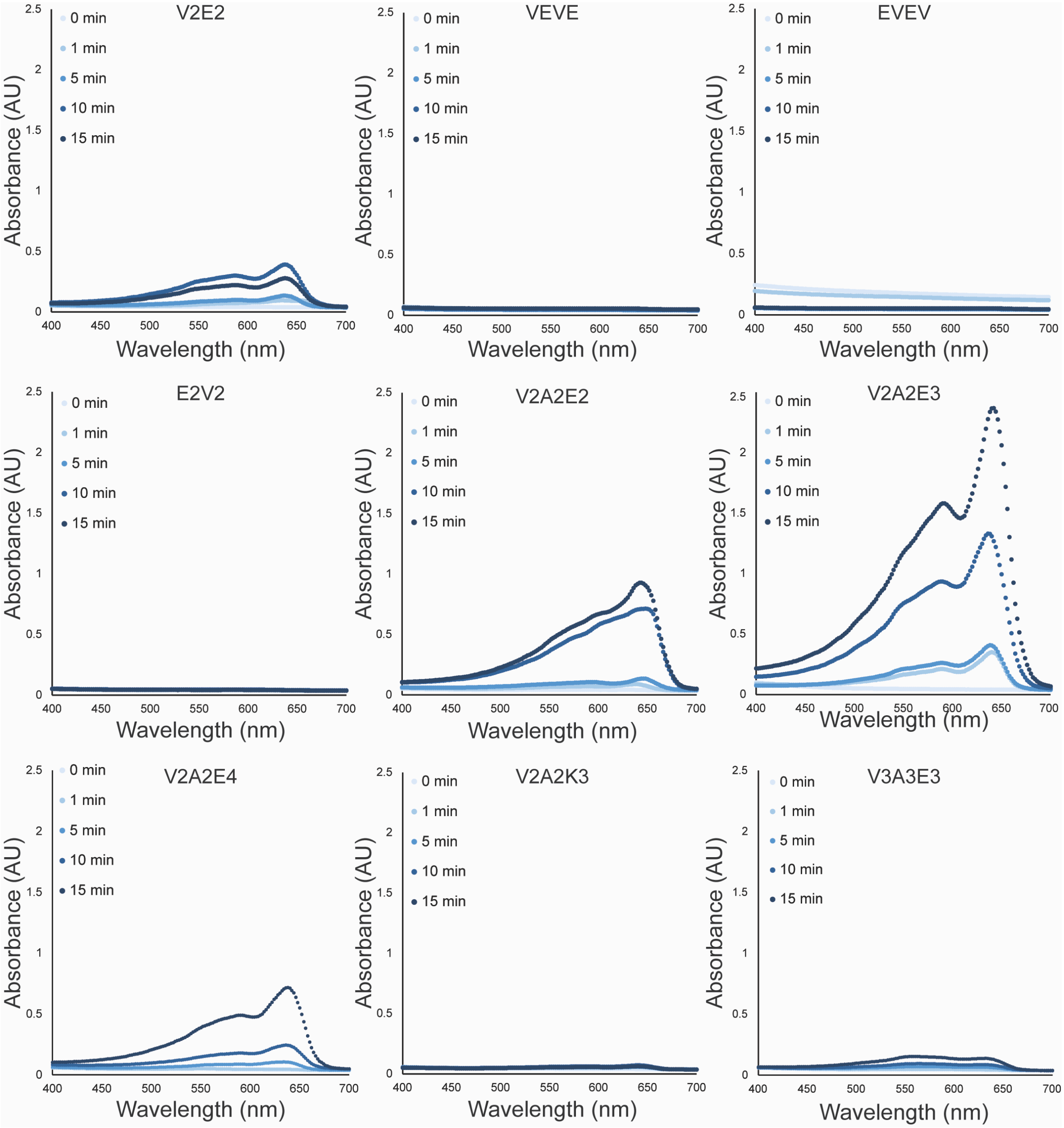

Figure S1. Quantification of DA-PA polymerization rates by UV-Vis.

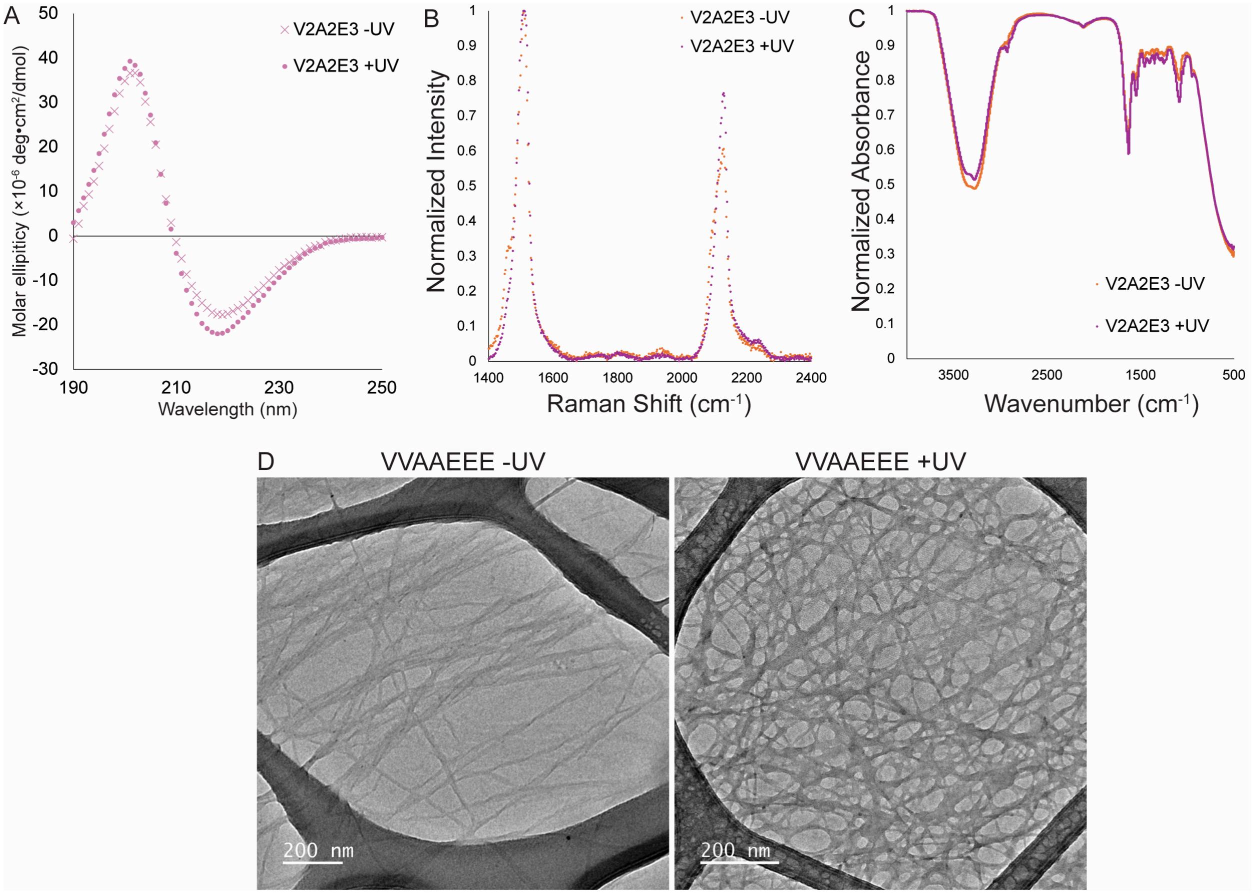

Figure S2. (A) Circular dichroism of the V2A2E3 DA-PA before and after UV polymerization. (B) Raman scattering of the V2A2E3 DA-PA before and after UV polymerization. (C) FTIR of the V2A2E3 DA-PA before and after UV polymerization.(D) TEM of the V2A2E3 DA-PA before and after UV polymerization.

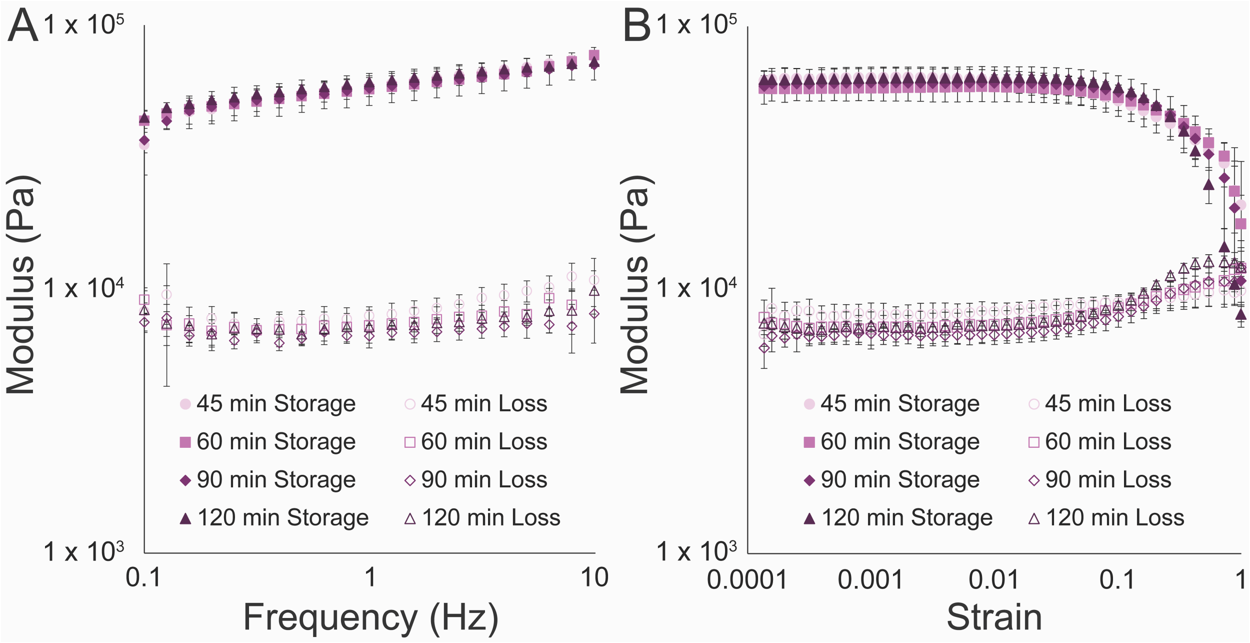

Figure S3. 8-arm PEG hydrogels containing the V3A3E3 DA-PA had minimal changes in hydrogel mechanical properties after 45 minutes of polymerization time as a function of (A) frequency or (B) strain.

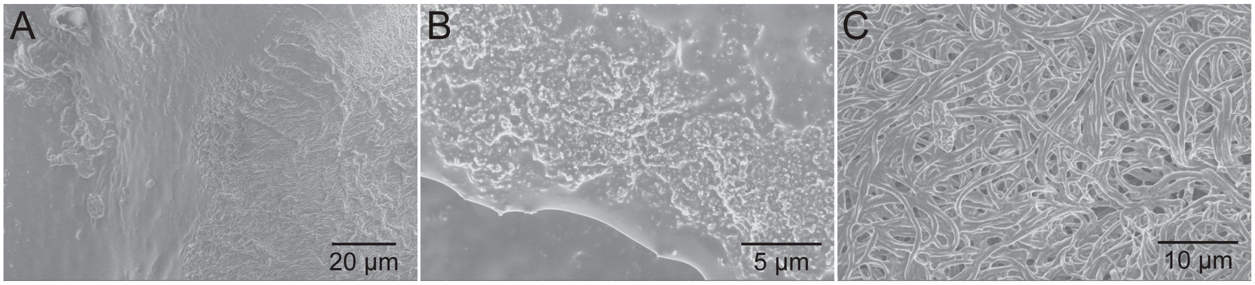

Figure S4. Scanning electron microscopy was performed on (A) a PEG hydrogel without DA-PA, (B) a PEG hydrogel with V2A2E3 DA-PAs prior to UV polymerization, and (C) a V2A2E3 DA-PA hydrogel after UV polymerization. Notably, the presence of high aspect ratio nanofibers visible within the electron microscopy after UV polymerization.

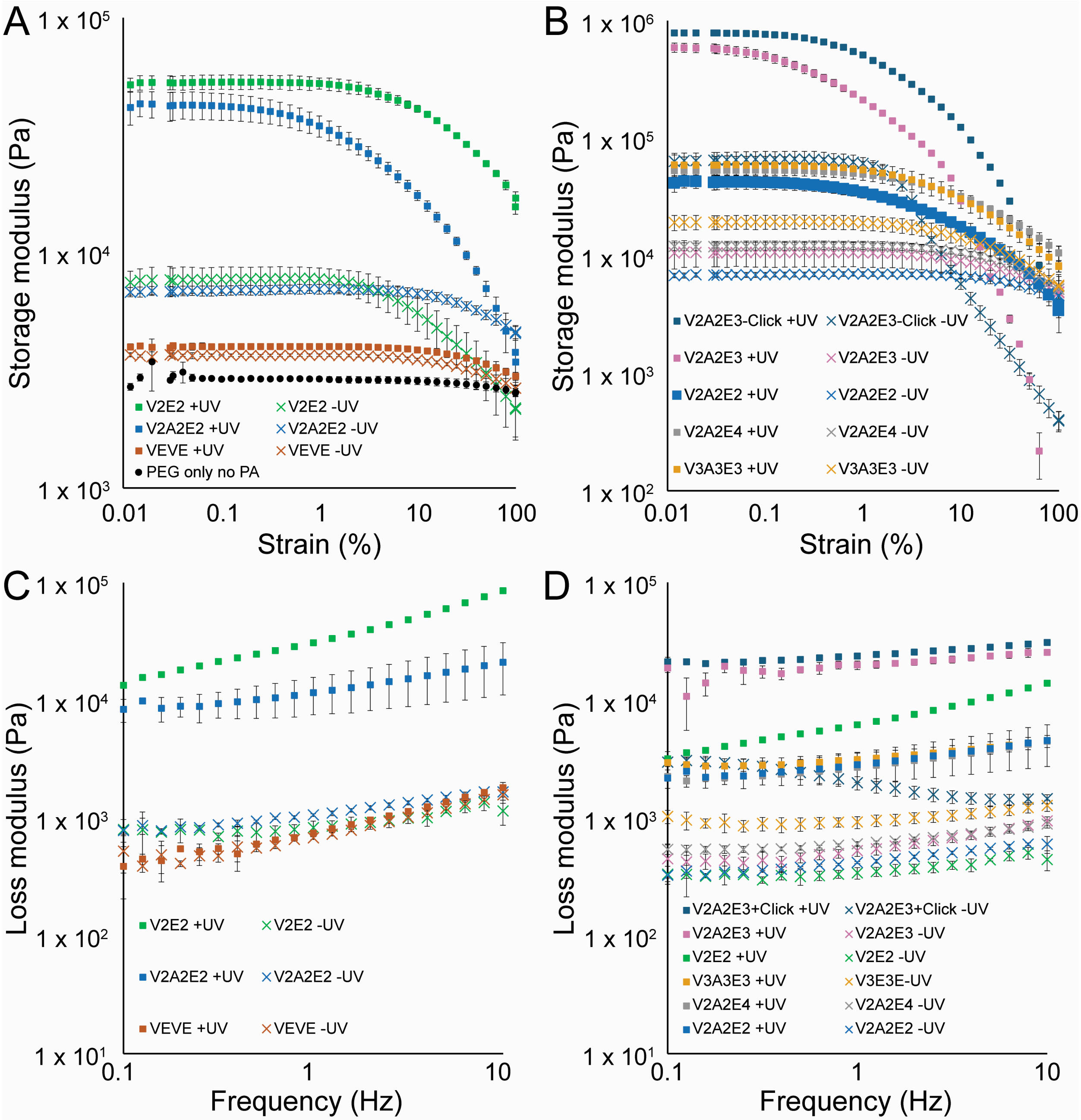

A
1 x 10^5
1 x 10^4
1 x 10^3
Storage modulus (Pa)
V2E2 +UV
V2E2 -UV
V2A2E2 +UV
V2A2E2 -UV
VEVE +UV
VEVE -UV
PEG only no PA
0.01
0.1
1
10
100
Strain (%)
B
1 x 10^6
1 x 10^5
1 x 10^4
1 x 10^3
1 x 10^2
Storage modulus (Pa)
V2A2E3-Click +UV
V2A2E3-Click -UV
V2A2E3 +UV
V2A2E3 -UV
V2A2E2 +UV
V2A2E2 -UV
V2A2E4 +UV
V2A2E4 -UV
V3A3E3 +UV
V3A3E3 -UV
0.01
0.1
1
10
100
Strain (%)
C
1 x 10^5
1 x 10^4
1 x 10^3
1 x 10^2
1 x 10^1
Loss modulus (Pa)
V2E2 +UV
V2E2 -UV
V2A2E2 +UV
V2A2E2 -UV
VEVE +UV
VEVE -UV
0.1
1
10
Frequency (Hz)
D
1 x 10^5
1 x 10^4
1 x 10^3
1 x 10^2
1 x 10^1
Loss modulus (Pa)
V2A2E3+Click +UV
V2A2E3+Click -UV
V2A2E3 +UV
V2A2E3 -UV
V2E2 +UV
V2E2 -UV
V3A3E3 +UV
V3E3E-UV
V2A2E4 +UV
V2A2E4 -UV
V2A2E2 +UV
V2A2E2 -UV
0.1
1
10
Frequency (Hz)

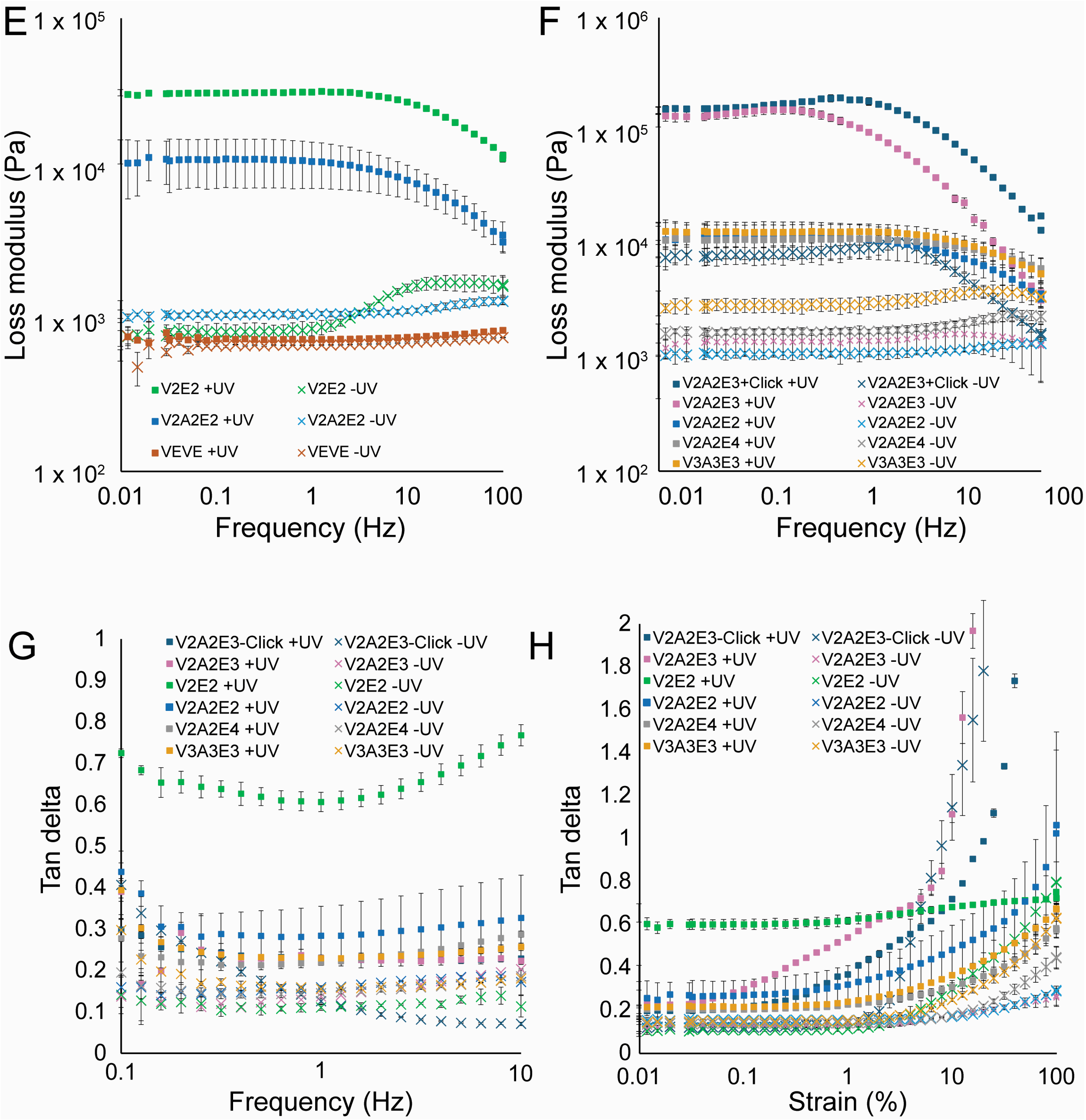

Figure S5. Rheometry of DA-PA hydrogels. The storage modulus of the hydrogels was quantified for the hydrogels as a function of strain for (A) selected peptides in the initial screen and (B) peptides derived from the V2A2E3 sequence. The loss modulus was also quantified a function of frequency for (C) selected peptides in the initial screen and (D) peptides derived from the V2A2E3 sequence. The loss modulus was quantified a function of strain for (E) selected peptides in the initial screen and (F) peptides derived from the V2A2E3 sequence. The tan δ was quantified for (G) frequency and (H) strain. Each data point is the average of three runs, and the error bars represent ± standard deviation.

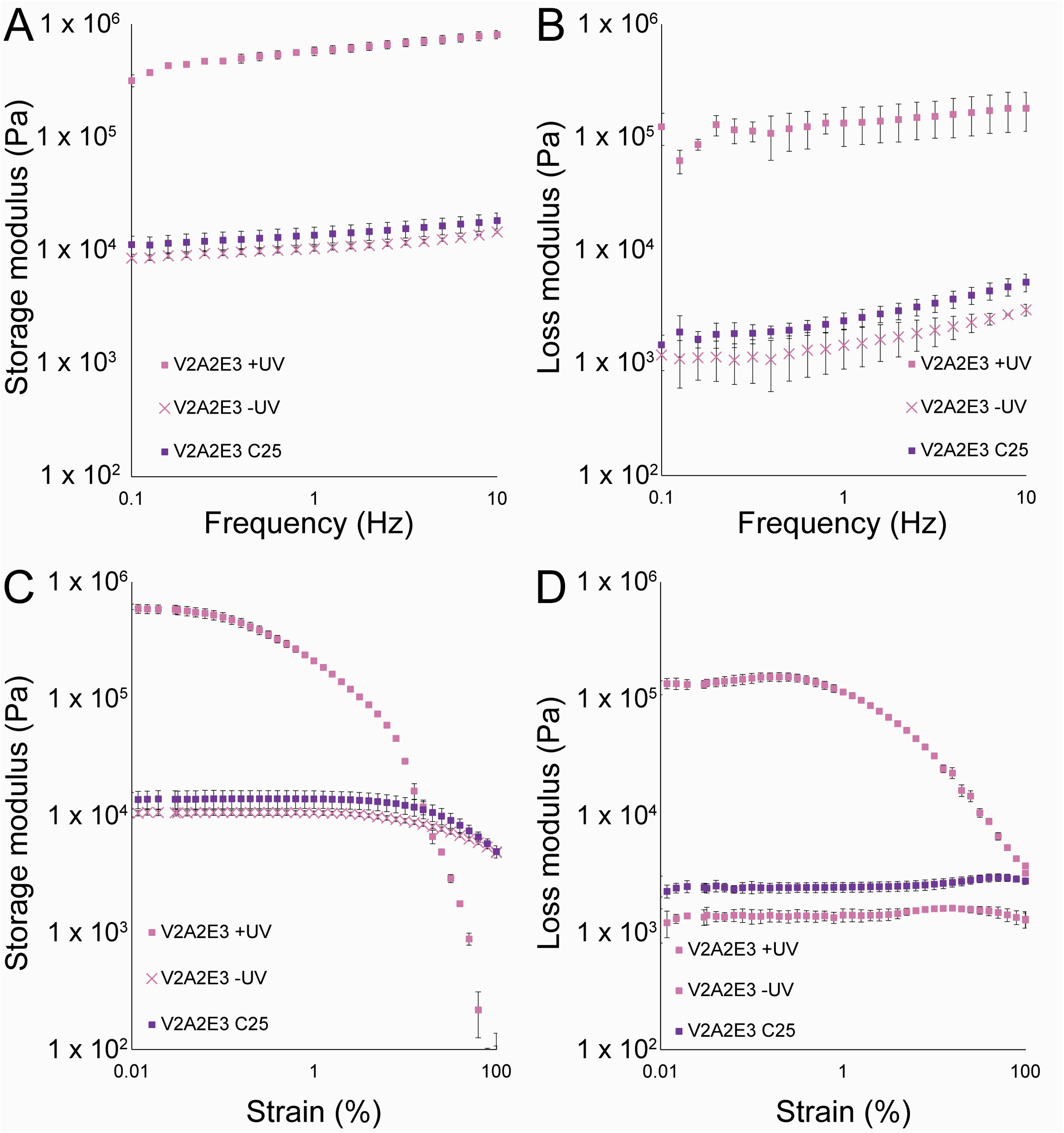

Figure S6. The effect of covalent polymerization on hydrogel mechanics was probed using a diacetylene tail both without (-UV) and with (+UV) UV polymerization. Additionally, a C25 alkyl carbon tail that lacked the diacetylene moieties was used. Hydrogels with the C25 tail and the DA tail without UV have similar mechanical properties, while UV polymerized hydrogels had a dramatic increase in storage and loss moduli. Hydrogels were studied at different frequencies for (A) the storage modulus and (B) the loss modulus, and different strains for (C) the storage modulus and (D) the loss modulus. Each data point is the average of three runs, and the error bars represent ± standard deviation.

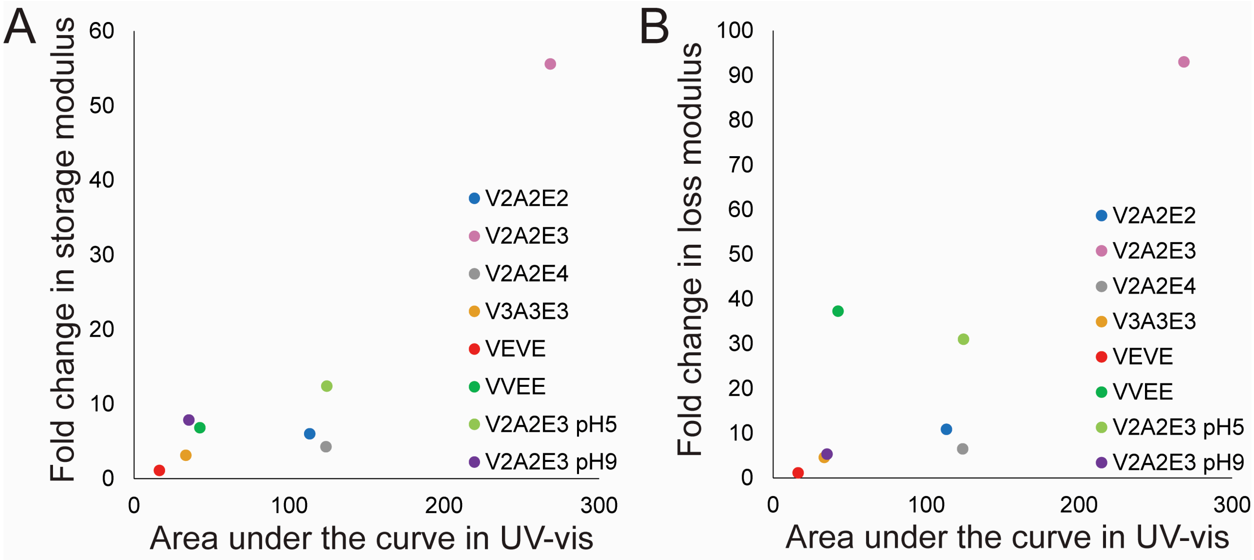

Figure S7. Comparison of the change in mechanical properties for DA-PA hydrogels upon polymerization compared to the propensity for polymerization, as quantified by the area under the curve in UV-Vis spectroscopy. Mechanical properties were quantified for the (A) storage and (B) loss moduli.

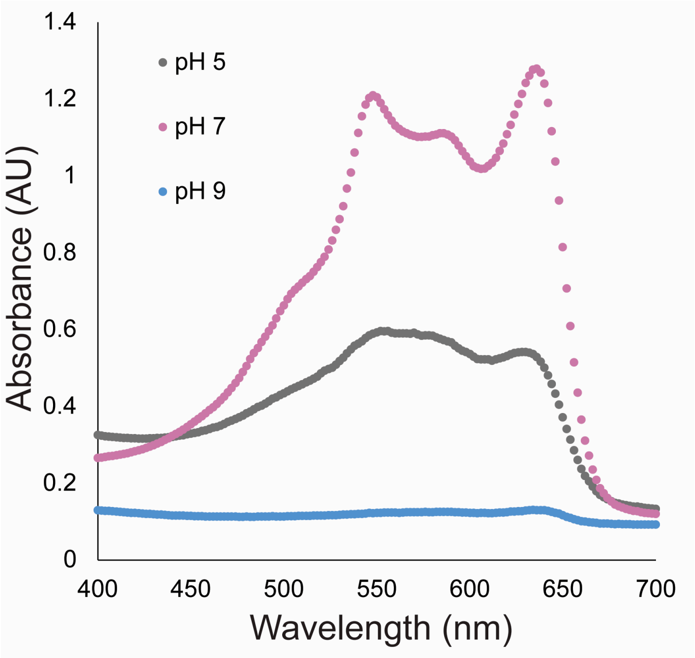

Figure S8. V2A2E3 DA-PA was polymerized at different pH values and the absorbance was quantified after 15 minutes. The ability of the V2A2E3 DA-PA was found to be strongly dependent on pH, with maximal polymerization at pH 7, significantly reduced polymerization at pH 5, and minimal polymerization at pH9.

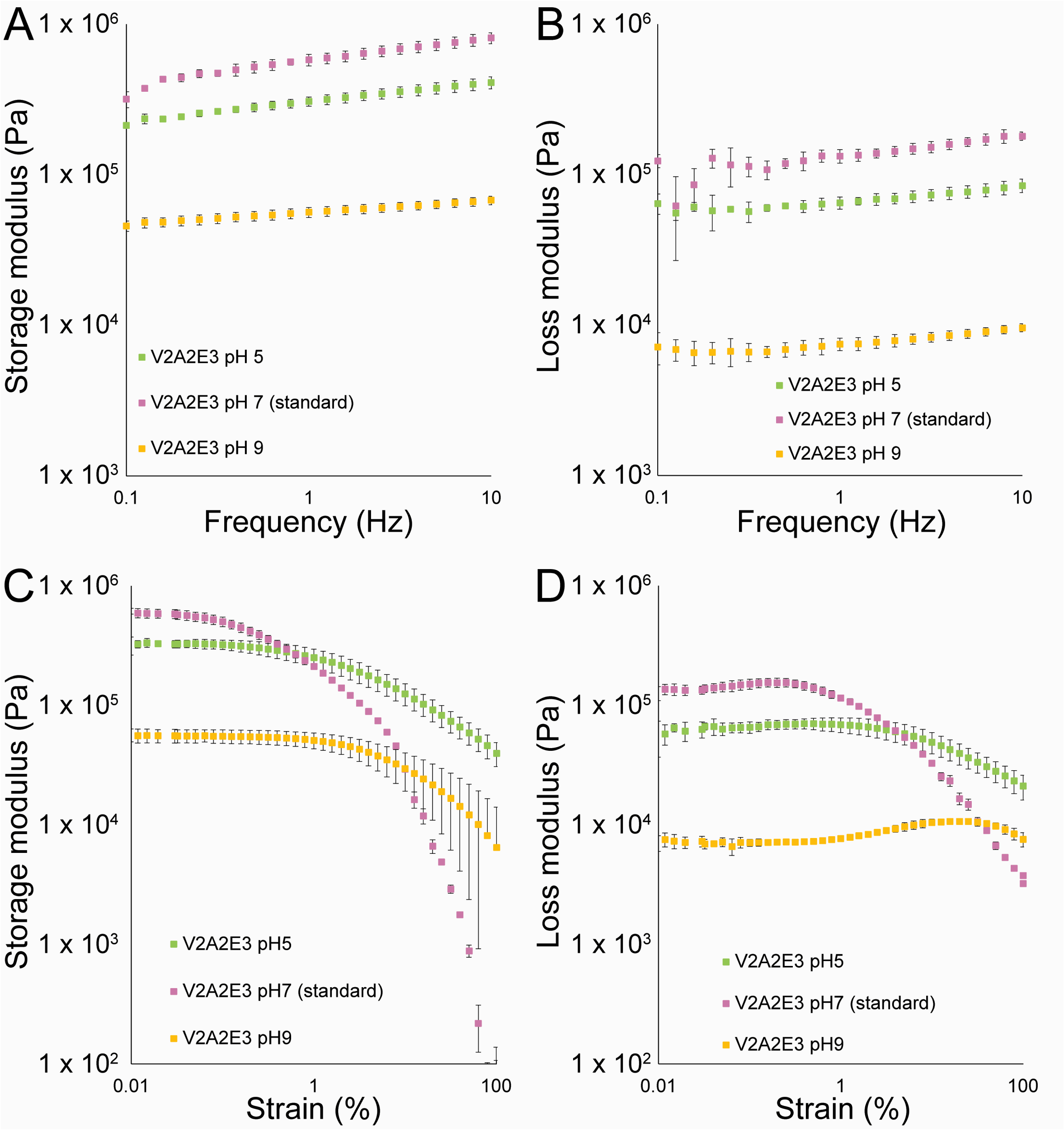

Figure S9. Rheometry of DA-PA hydrogels at different polymerization pH values, including acidic pH (5), neutral pH (7), and basic pH (9). Hydrogels were studied at different frequencies for (A) the storage modulus and (B) the loss modulus, and different strains for (C) the storage modulus and (D) the loss modulus. Each data point is the average of three runs, and the error bars represent ± standard deviation.

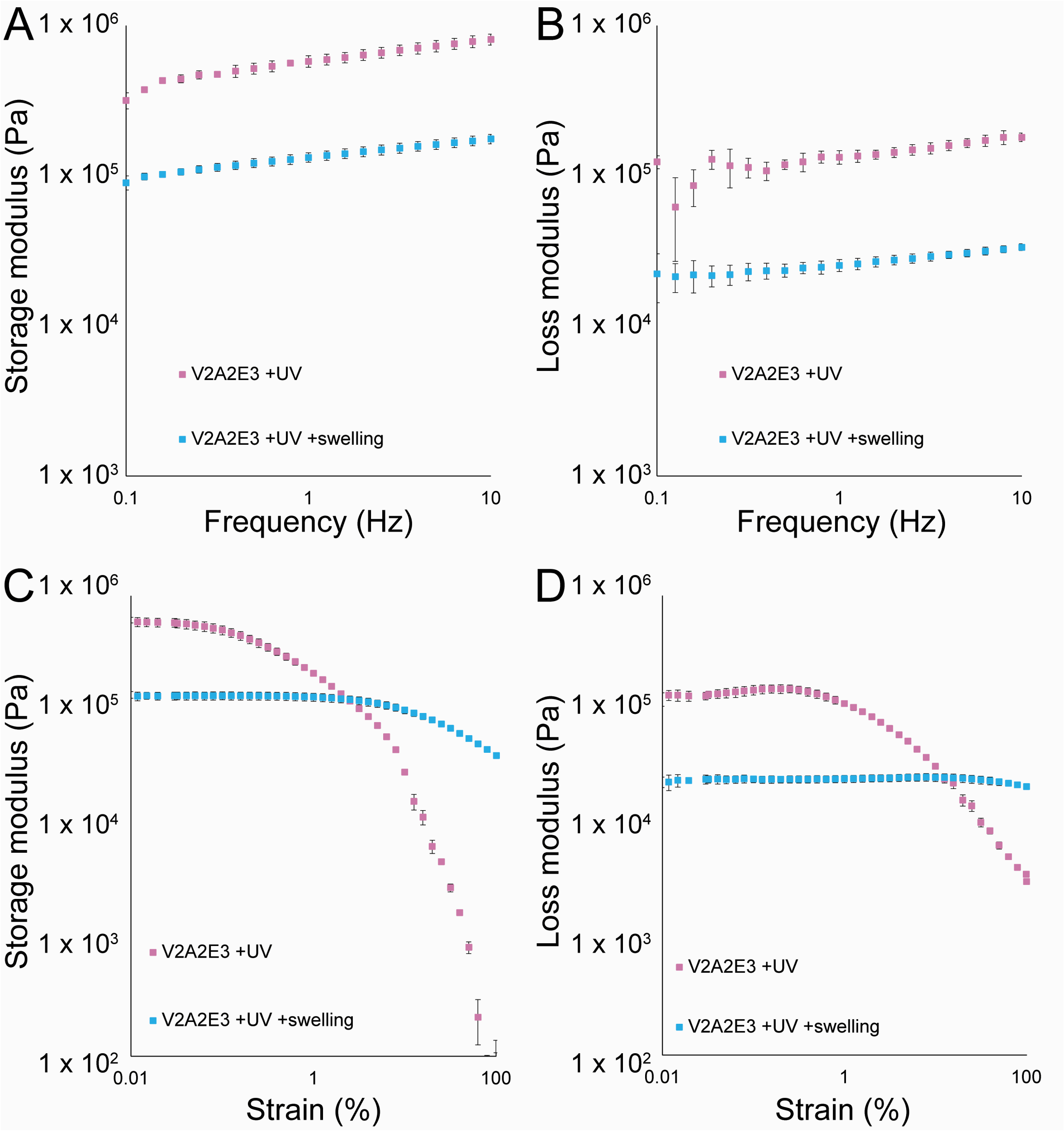

Figure S10. The effect of swelling on hydrogel mechanics was studied by incubating the V2A2E3 DA-PA gels in 1X PBS at 37 °C for 24 hours after UV polymerization. Hydrogels were studied at different frequencies for (A) the storage modulus and (B) the loss modulus, and different strains for (C) the storage modulus and (D) the loss modulus. Each data point is the average of three runs, and the error bars represent ± standard deviation.

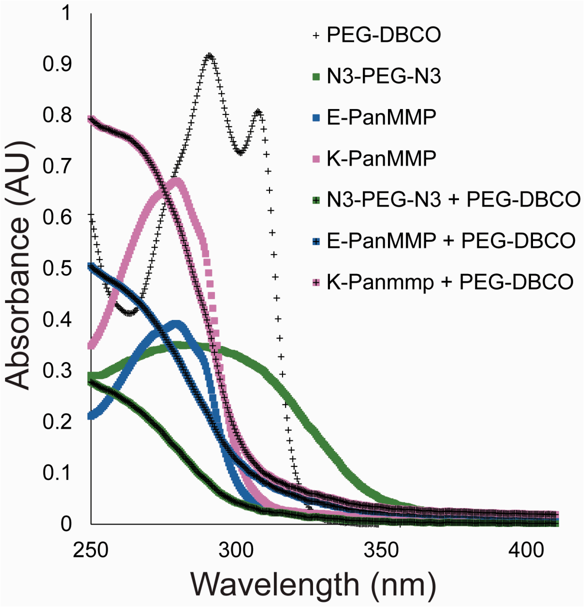

Figure S11. The reactivity of PEG-DBCO with the PEG crosslinker (N3-PEG-N3) and both positively (K-PanMMP) and negatively (E-PanMMP) charged peptides was quantified after 10 minutes with UV-vis spectroscopy. The characteristic DBCO UV absorption bands disappear, indicating the DBCO has undergone nearly complete reaction after 10 minutes with all three peptides.

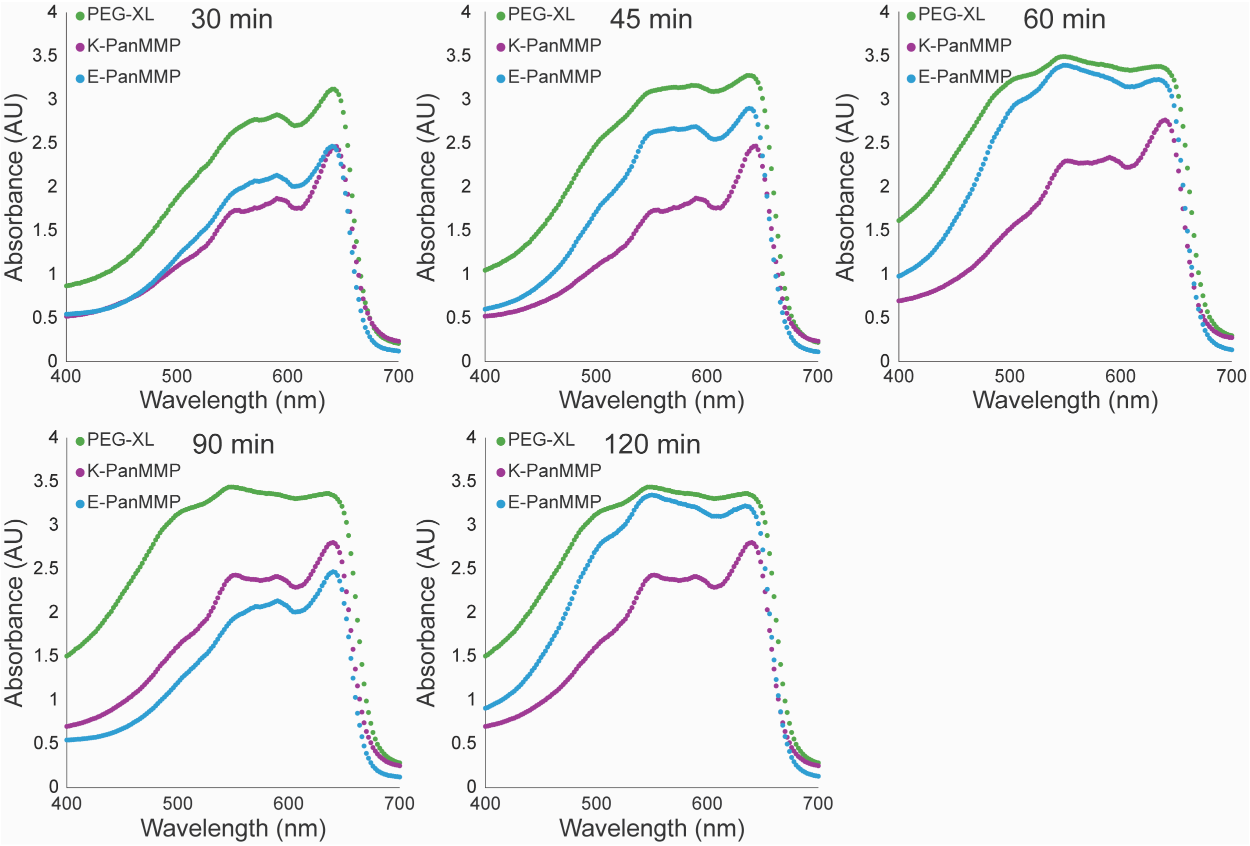

Figure S12. The diacetylene moiety on V2A2E3 peptide amphiphile was able to polymerize in PEG hydrogels crosslinked by PEG (PEG-XL), a positively charged peptide (K-PanMMP), and a negatively charged peptide (E-PanMMP).

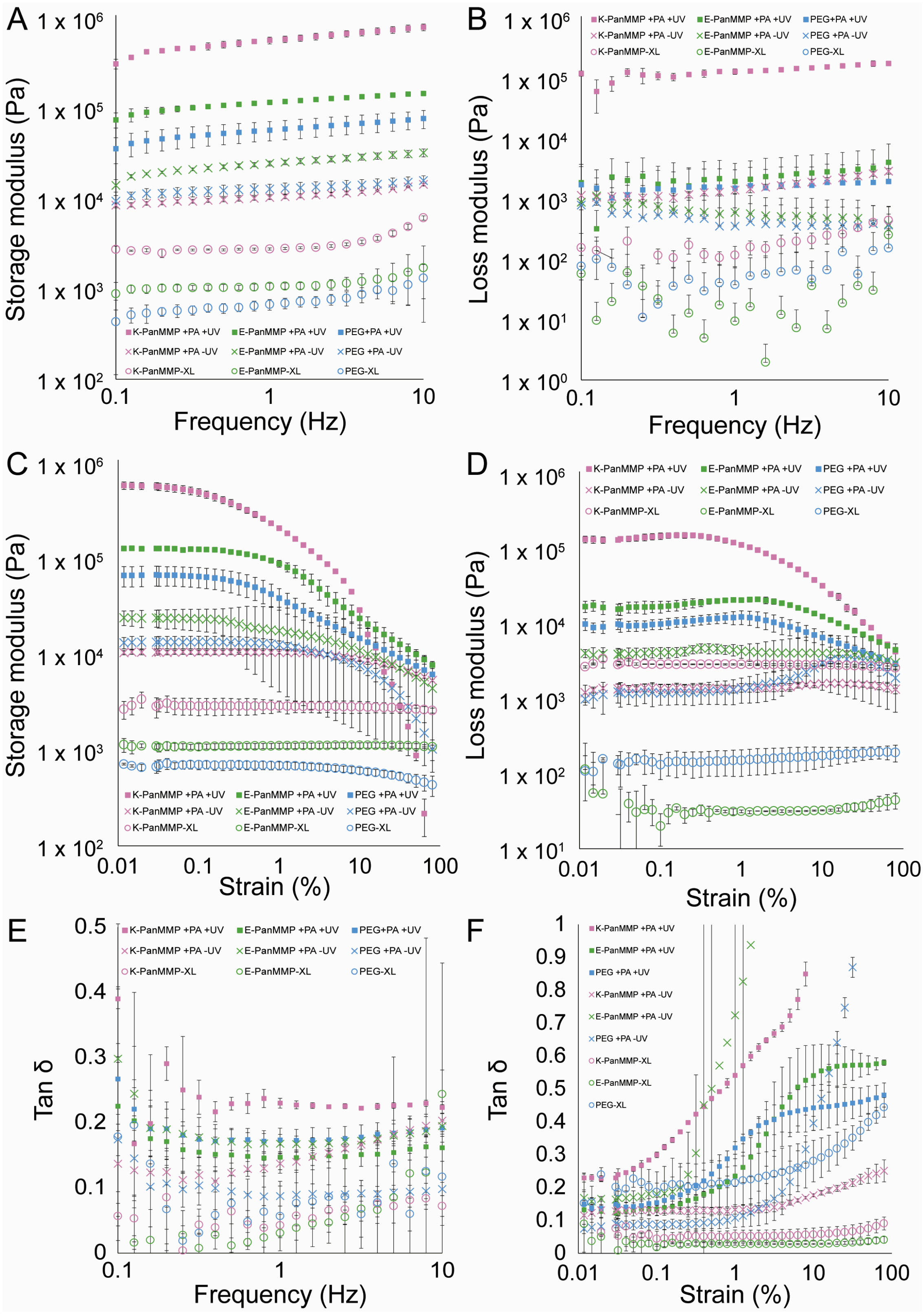

Figure S13. The viscoelastic properties of V2A2E3 IPN hydrogels with PEG, E-PanMMP and K-PanMMP crosslinkers. Frequency sweeps including the (A) storage modulus and (B) loss modulus, and strain sweeps including the (C) storage modulus and (D) loss modulus. The tan δ values for (E) frequency and (F) strain sweeps. Error bars represent ± standard deviation. N = 3. Statistical analyses can be found in the supporting information.

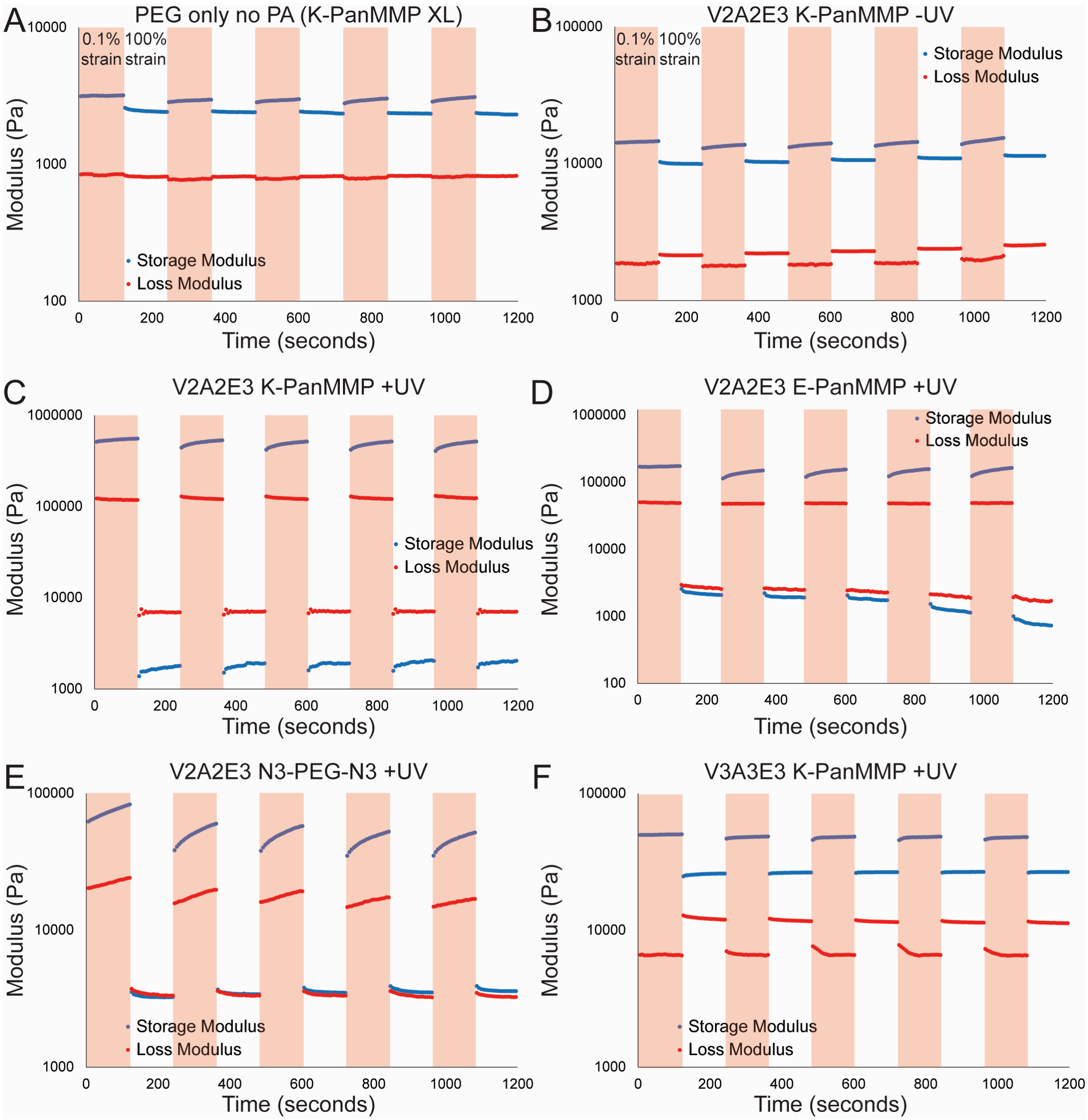

Figure S14. Cyclic strain studies were performed to understand how DA-PA hydrogels recovered after undergoing significant strains. Hydrogels underwent alternating 2 minute cycles of low 0.1% strains (shaded regions) and high 100% strains (unshaded regions) and the rheological properties were measured at 1 Hz. Test were performed on (A) K-PanMMP hydrogels without DA-PAs (B) unpolymerized V2A2E3 DA-PA in K-PanMMP crosslinked PEG networks, (C) polymerized V2A2E3 DA-PA in K-PanMMP crosslinked PEG networks, (D) polymerized V2A2E3 DA-PA in E-PanMMP crosslinked PEG networks, (E) polymerized V2A2E3 DA-PA in PEG crosslinked PEG networks, and (F) polymerized V3A3E3 DA-PA in K-PanMMP crosslinked PEG networks Each trace is the average of three runs.

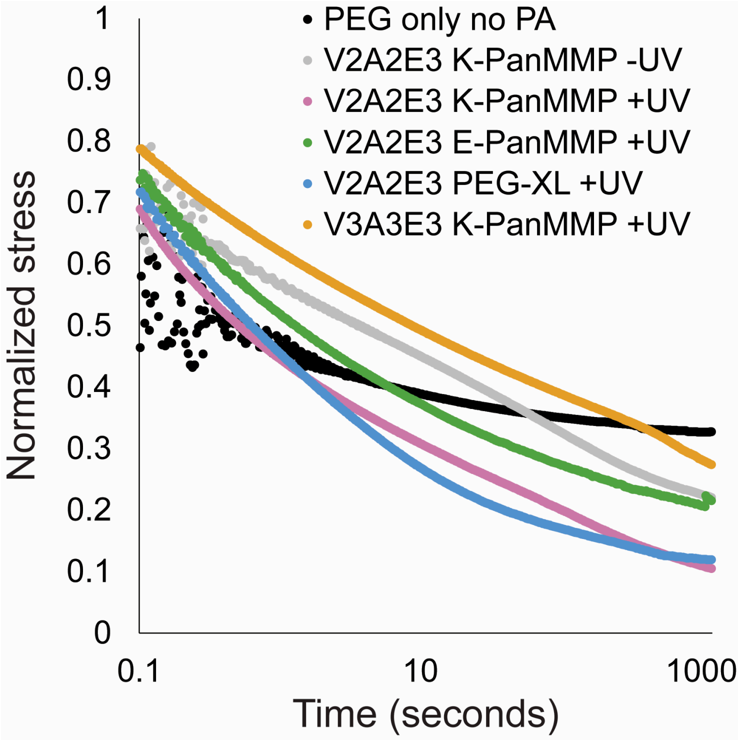

Figure S15. Stress relaxation behavior of DA-PA hydrogels was determined by placing a 10% strain on the hydrogels and measuring the change in normalized stresses over time.

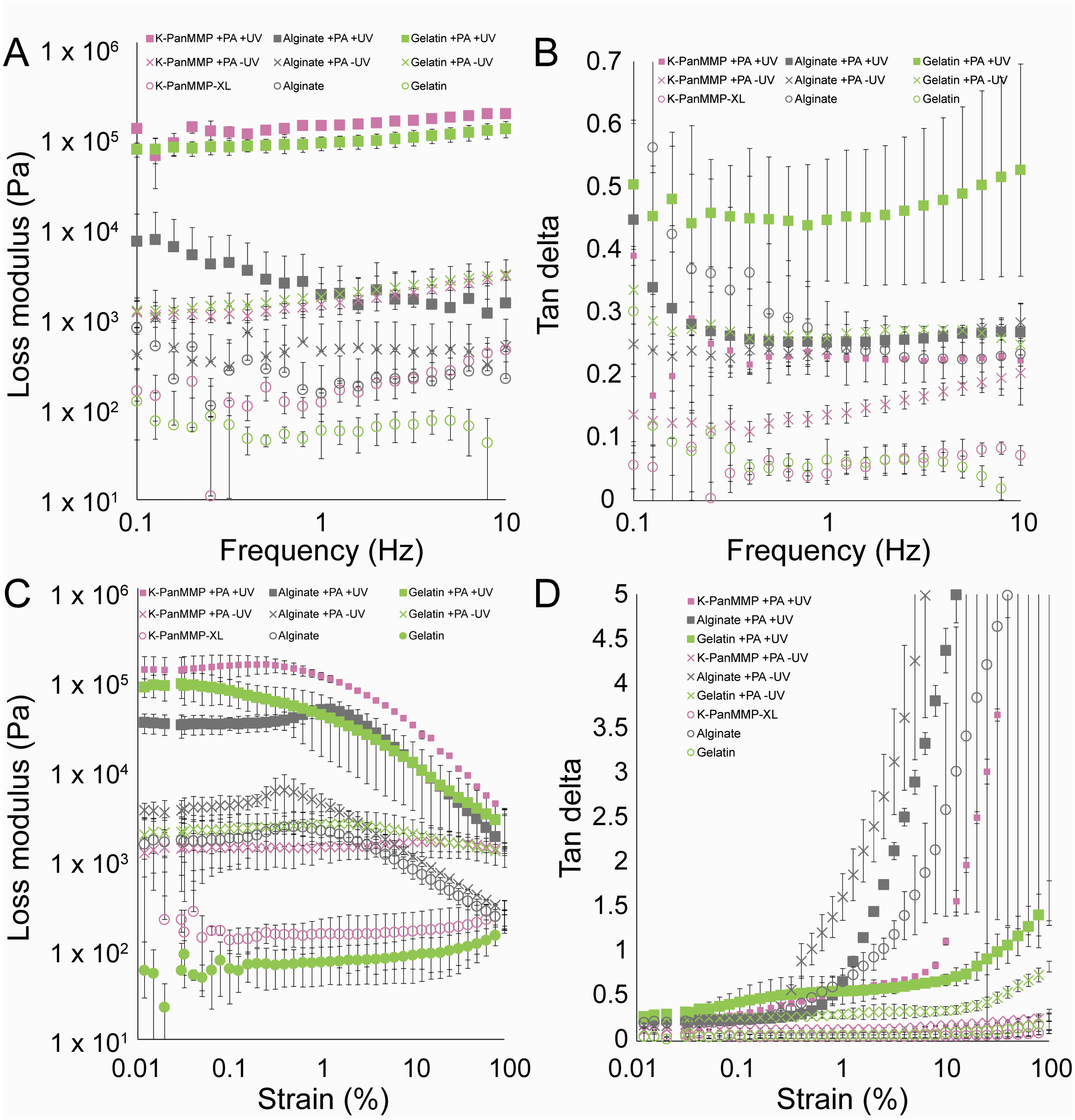

Figure S16. The viscoelastic properties of V2A2E3 IPN hydrogels with PEG, alginate and gelatin. The PEG gel was crosslinked with the K-PanMMP peptide. Frequency sweeps including the (A) loss modulus and (B) tan δ, and strain sweeps including the (C) loss modulus and (D) tan δ. Error bars represent ± standard deviation, N = 3. Statistical analyses can be found further down in the supporting information.

A

10,12-Pentacosadiynoyl-$V_2A_2E_2$-NH_2

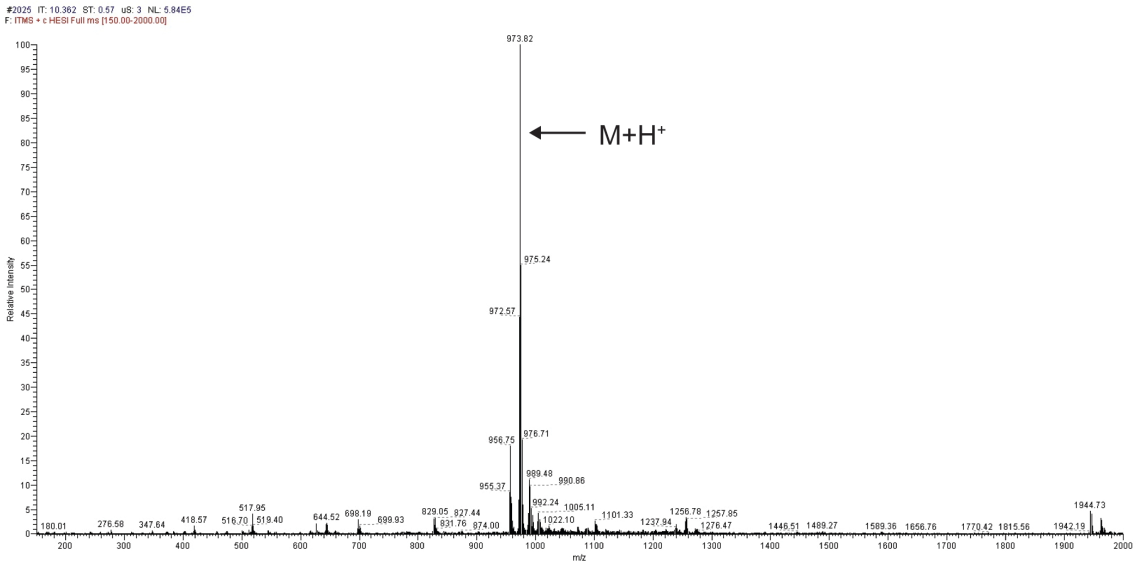

B

10,12-Pentacosadiynoyl-$V_2A_2E_3$-NH_2

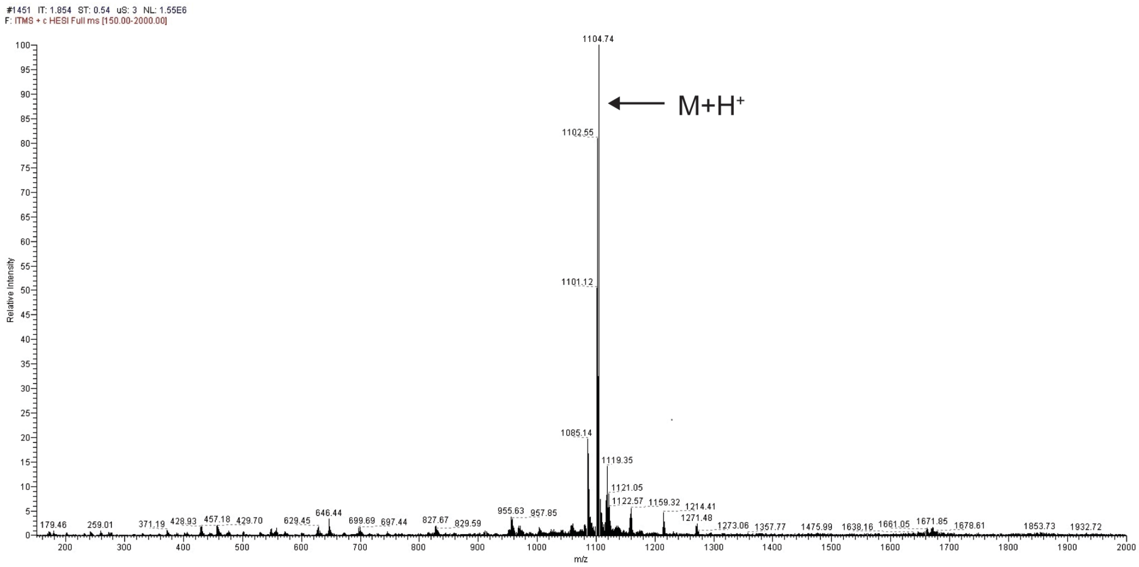

C

10,12-Pentacosadiynoyl-$V_2A_2E_4$-NH_2

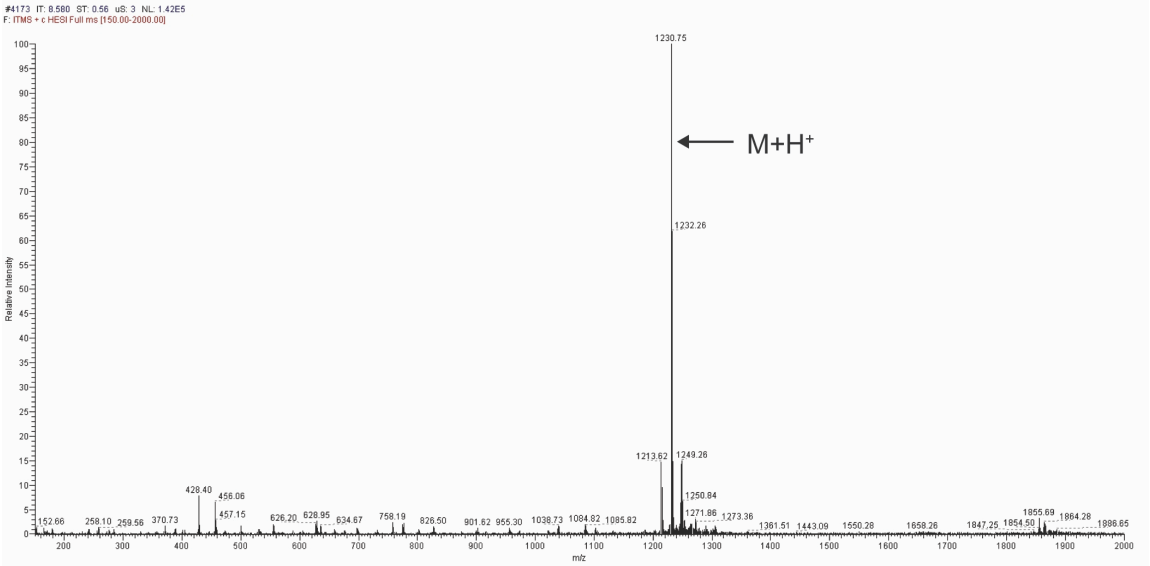

D

10,12-Pentacosadiynoyl-$V_3A_3E_3$-NH_2

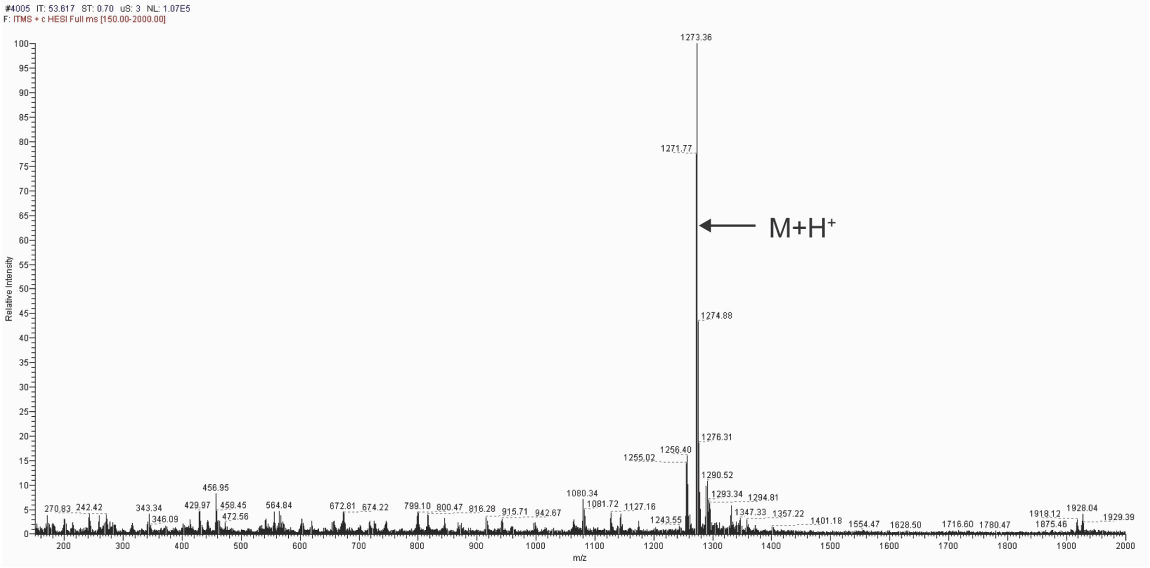

E

10,12-Pentacosadiynoyl-$V_2A_2E_3K(N_3)$-NH_2

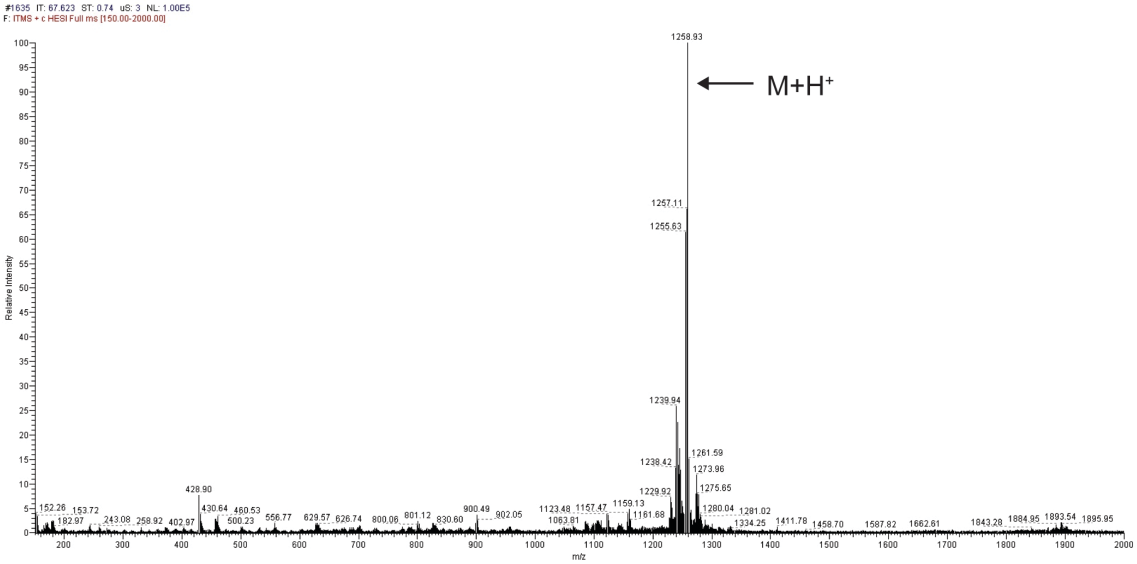

F

10,12-Pentacosadiynoyl-E_2V_2-NH_2

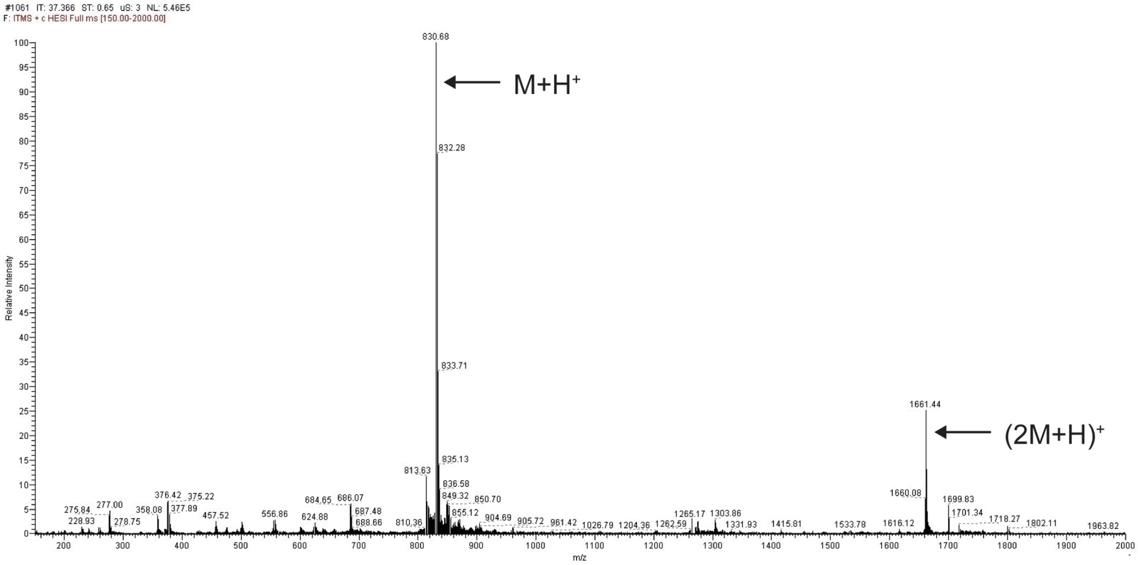

G

10,12-Pentacosadiynoyl-EVEV-NH_2

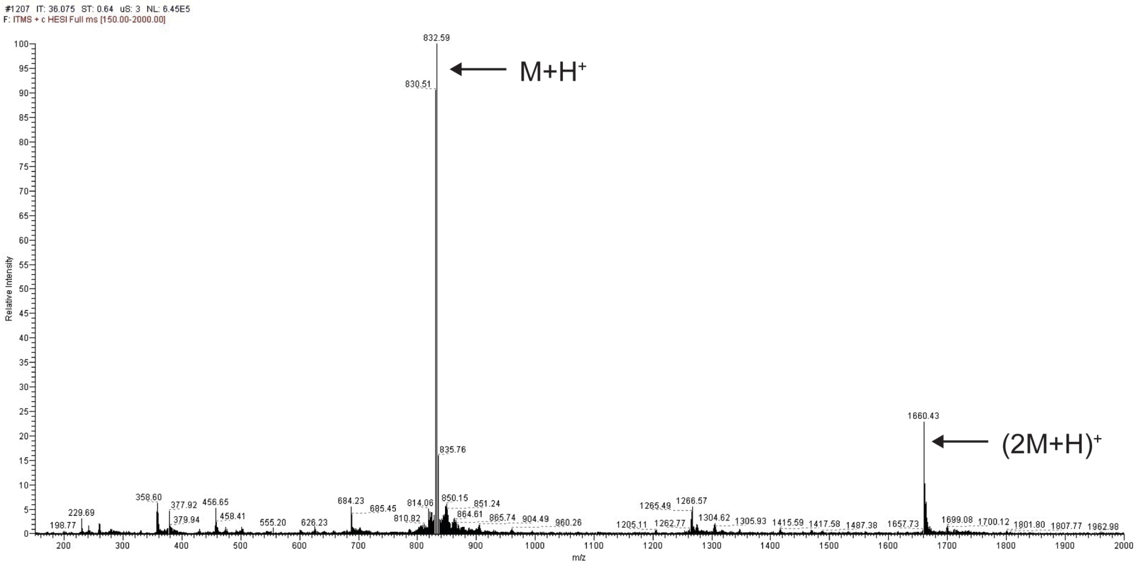

H

10,12-Pentacosadiynoyl-VEVE-NH_2

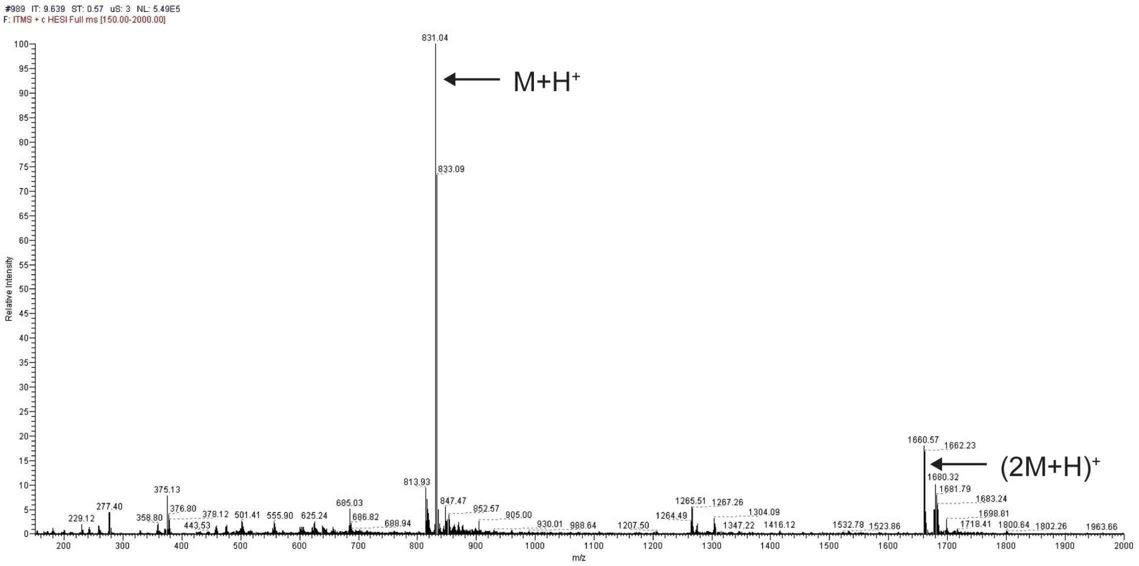

I

10,12-Pentacosadiynoyl-V_2E_2-NH_2

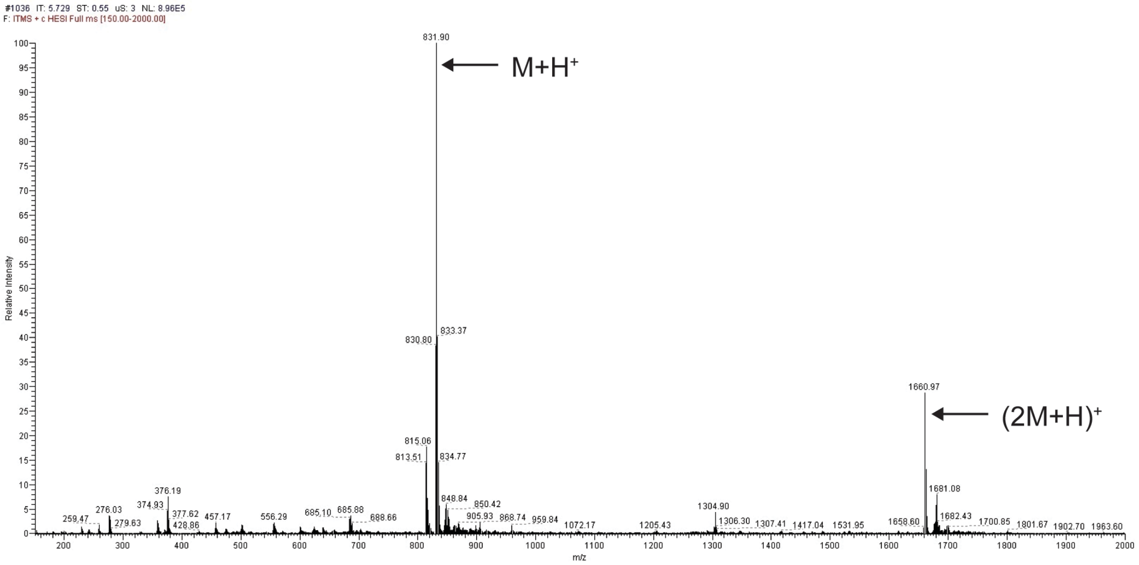

J

10,12-Pentacosadiynoyl-$V_2A_2K_3$-NH_2

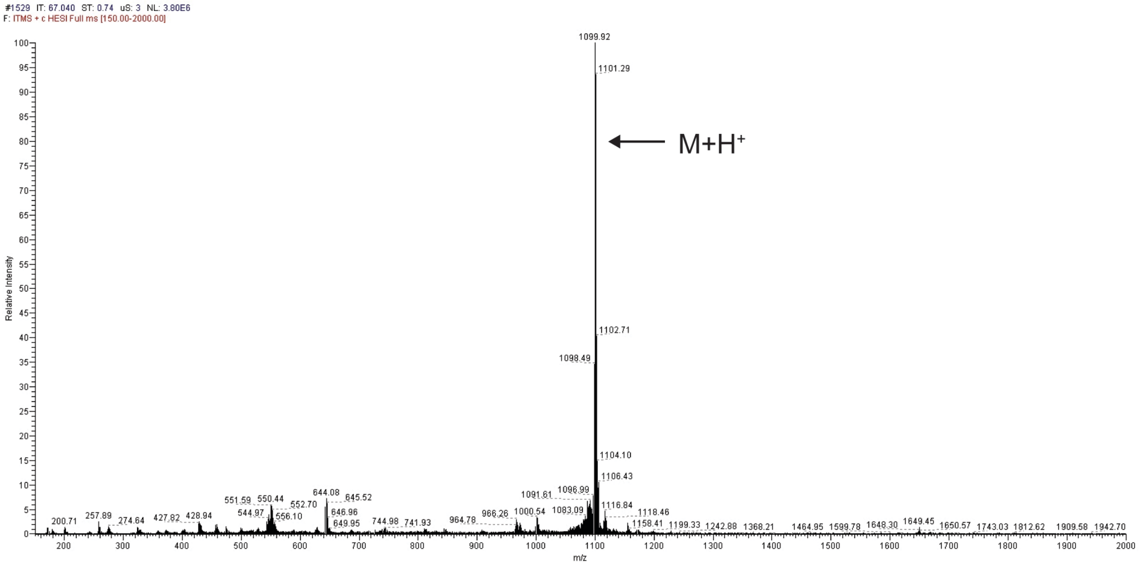

K

Pentacosanoic-VVAAEEE-NH_2

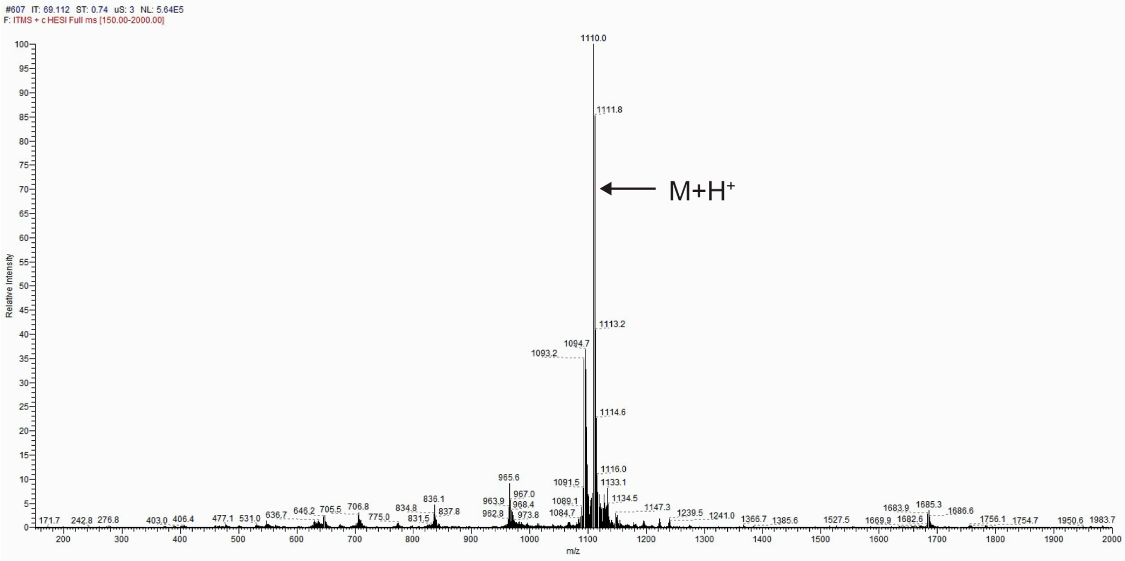

L

N_3-Gly-KGPQGIWGQKK(N_3)-NH_2

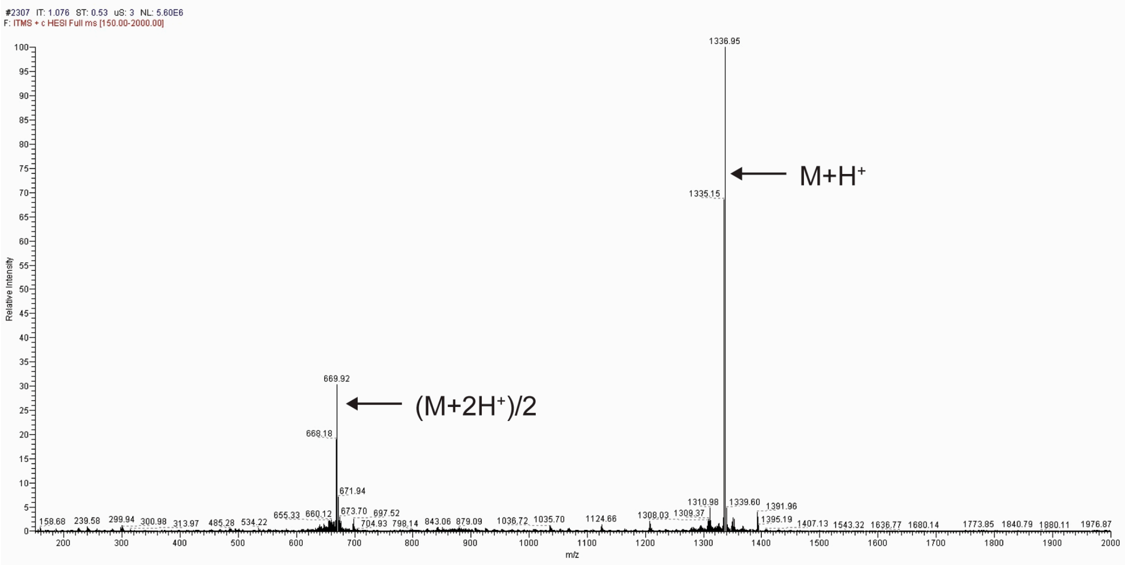

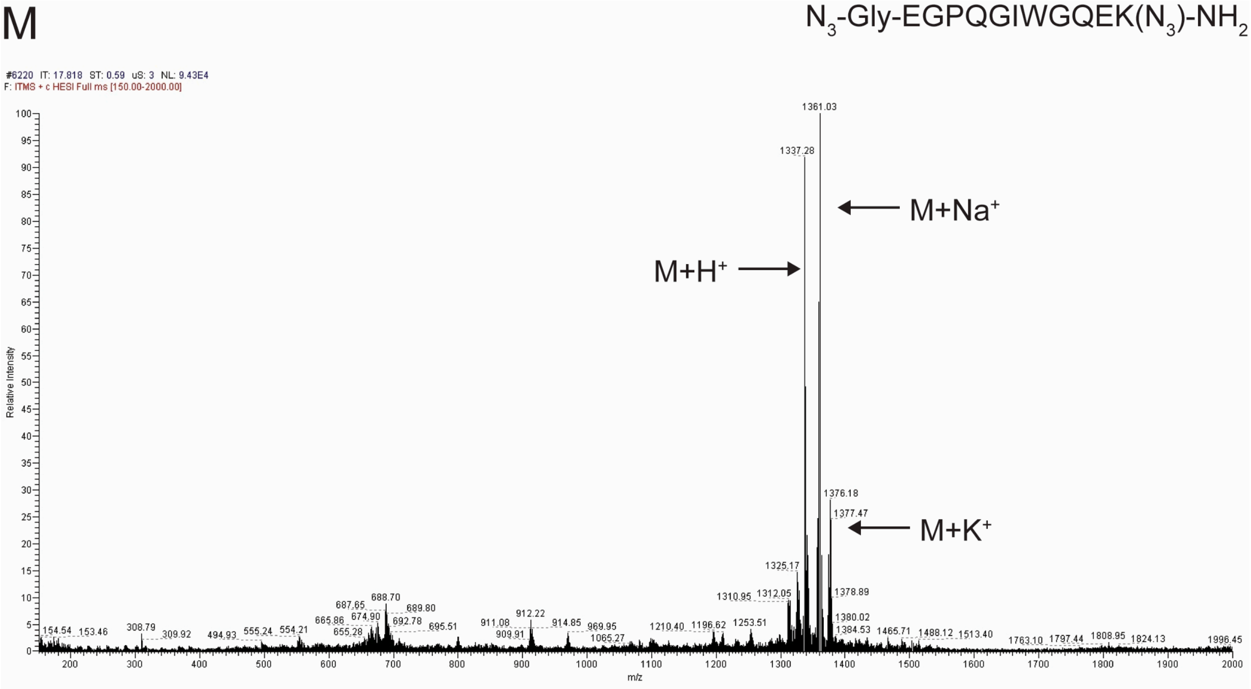

Figure S17. Mass spectrometry characterization of the peptides used in this work. (A) $V_2A_2E_2$, (B) $V_2A_2E_3$, (C) $V_2A_2E_4$, (D) $V_3A_3E_3$, (E) $V_2A_2E_3K(N_3)$, (F) E2V3, (G) EVEV, (H) VEVE, (I) V_2E_2, (J) $V_2A_2K_3$, (K) C25-V2A2E3, (L) N_3-KGPQGIWGQK-K(N_3), and (M) N_3-EGPQGIWGQE-K(N_3).

Statistical Analysis:
Across all conditions we ran ANOVAs followed by Tukey post-hoc tests. The pair-wise comparisons from the Tukey test are found below. The moduli values from the frequency sweeps at 1 Hz were used to calculate the tan δ values and all statistics, and an $N = 3$ was used for all conditions.

Initial rheological comparison (Figure 4D/S4): storage modulus					
**Condition 1**	**Condition 2**	**Estimate**	**Conf.low**	**Conf.high**	**Adj.p.value**
V2A2E2 -UV	PEG only (no DA-PA)	3895	-3041.6712	10831.6712	0.50100518
V2A2E2 +UV	PEG only (no DA-PA)	37371.6667	30434.9955	44308.3378	5.94E-10
V2E2 -UV	PEG only (no DA-PA)	4699.33333	-2237.3378	11636.0045	0.30266291
V2E2 +UV	PEG only (no DA-PA)	48128.3333	41191.6622	55065.0045	2.10E-11
VEVE -UV	PEG only (no DA-PA)	792.32	-6144.3512	7728.99118	0.99961542
VEVE +UV	PEG only (no DA-PA)	1155.49667	-5781.1745	8092.16784	0.99681661
V2A2E2 +UV	V2A2E2 -UV	33476.6667	26539.9955	40413.3378	2.40E-09
V2E2 -UV	V2A2E2 -UV	804.333333	-6132.3378	7741.00451	0.99958082
V2E2 +UV	V2A2E2 -UV	44233.3333	37296.6622	51170.0045	7.27E-11
VEVE -UV	V2A2E2 -UV	-3102.68	-10039.351	3833.99118	0.72573698
VEVE +UV	V2A2E2 -UV	-2739.5033	-9676.1745	4197.16784	0.81868768
V2E2 -UV	V2A2E2 +UV	-32672.333	-39609.005	-25735.662	3.32E-09
V2E2 +UV	V2A2E2 +UV	10756.6667	3819.99549	17693.3378	0.00168738
VEVE -UV	V2A2E2 +UV	-36579.347	-43516.018	-29642.675	7.72E-10
VEVE +UV	V2A2E2 +UV	-36216.17	-43152.841	-29279.499	8.73E-10
V2E2 +UV	V2E2 -UV	43429	36492.3288	50365.6712	9.31E-11
VEVE -UV	V2E2 -UV	-3907.0133	-10843.685	3029.65784	0.49767745
VEVE +UV	V2E2 -UV	-3543.8367	-10480.508	3392.83451	0.60074794
VEVE -UV	V2E2 +UV	-47336.013	-54272.685	-40399.342	2.72E-11
VEVE +UV	V2E2 +UV	-46972.837	-53909.508	-40036.165	3.06E-11
VEVE +UV	VEVE -UV	363.176667	-6573.4945	7299.84784	0.99999602

Initial rheological comparison (Figure 4D/S4): loss modulus					
**condition 1**	**condition 2**	**estimate**	**conf.low**	**conf.high**	**adj.p.value**

V2A2E2 -UV	PEG only (no DA-PA)	947.186667	-3446.8045	5341.17787	0.98757072
V2A2E2 +UV	PEG only (no DA-PA)	11524.8533	7130.86213	15918.8445	5.98E-06
V2E2 -UV	PEG only (no DA-PA)	708.786667	-3685.2045	5102.77787	0.9973289
V2E2 +UV	PEG only (no DA-PA)	30714.52	26320.5288	35108.5112	1.86E-11
VEVE -UV	PEG only (no DA-PA)	559.394333	-3834.5969	4953.38554	0.99928646
VEVE +UV	PEG only (no DA-PA)	624.616333	-3769.3749	5018.60754	0.99867278
V2A2E2 +UV	V2A2E2 -UV	10577.6667	6183.67546	14971.6579	1.64E-05
V2E2 -UV	V2A2E2 -UV	-238.4	-4632.3912	4155.59121	0.99999509
V2E2 +UV	V2A2E2 -UV	29767.3333	25373.3421	34161.3245	3.04E-11
VEVE -UV	V2A2E2 -UV	-387.79233	-4781.7835	4006.19887	0.99991354
VEVE +UV	V2A2E2 -UV	-322.57033	-4716.5615	4071.42087	0.99997063
V2E2 -UV	V2A2E2 +UV	-10816.067	-15210.058	-6422.0755	1.27E-05
V2E2 +UV	V2A2E2 +UV	19189.6667	14795.6755	23583.6579	9.27E-09
VEVE -UV	V2A2E2 +UV	-10965.459	-15359.45	-6571.4678	1.08E-05
VEVE +UV	V2A2E2 +UV	-10900.237	-15294.228	-6506.2458	1.16E-05
V2E2 +UV	V2E2 -UV	30005.7333	25611.7421	34399.7245	2.69E-11
VEVE -UV	V2E2 -UV	-149.39233	-4543.3835	4244.59887	0.9999997
VEVE +UV	V2E2 -UV	-84.170333	-4478.1615	4309.82087	0.99999999
VEVE -UV	V2E2 +UV	-30155.126	-34549.117	-25761.134	2.49E-11
VEVE +UV	V2E2 +UV	-30089.904	-34483.895	-25695.912	2.58E-11
VEVE +UV	VEVE -UV	65.222	-4328.7692	4459.21321	1

Initial rheological comparison (Figure 4D/S4): tan δ					
**condition 1**	**condition 2**	**estimate**	**conf.low**	**conf.high**	**adj.p.value**
V2A2E2 -UV	PEG only (no DA-PA)	0.11717	0.03600	0.19834	0.00322454

V2A2E2 +UV	PEG only (no DA-PA)	0.24158	0.16042	0.32275	1.29E-06
V2E2 -UV	PEG only (no DA-PA)	0.06800	-0.01316	0.14917	0.13003322
V2E2 +UV	PEG only (no DA-PA)	0.56404	0.48287	0.64520	2.05E-11
VEVE -UV	PEG only (no DA-PA)	0.14709	0.06593	0.22826	0.00036818
VEVE +UV	PEG only (no DA-PA)	0.14624	0.06507	0.22741	0.00039075
V2A2E2 +UV	V2A2E2 -UV	0.12442	0.04325	0.20558	0.00187808
V2E2 -UV	V2A2E2 -UV	-0.04916	-0.13033	0.03200	0.41896633
V2E2 +UV	V2A2E2 -UV	0.44687	0.36570	0.52803	4.57E-10
VEVE -UV	V2A2E2 -UV	0.02992	-0.05124	0.11109	0.85915799
VEVE +UV	V2A2E2 -UV	0.02907	-0.05210	0.11024	0.87397254
V2E2 -UV	V2A2E2 +UV	-0.17358	-0.25475	-0.09241	6.29E-05
V2E2 +UV	V2A2E2 +UV	0.32245	0.24128	0.40362	3.29E-08
VEVE -UV	V2A2E2 +UV	-0.09449	-0.17566	-0.01332	0.01816357
VEVE +UV	V2A2E2 +UV	-0.09535	-0.17651	-0.01418	0.01701223
V2E2 +UV	V2E2 -UV	0.49603	0.41486	0.57720	1.28E-10
VEVE -UV	V2E2 -UV	0.07909	-0.00208	0.16026	0.05838528
VEVE +UV	V2E2 -UV	0.07823	-0.00293	0.15940	0.06220543
VEVE -UV	V2E2 +UV	-0.41694	-0.49811	-0.33577	1.07E-09
VEVE +UV	V2E2 +UV	-0.41780	-0.49896	-0.33663	1.04E-09
VEVE +UV	VEVE -UV	-0.00085	-0.08202	0.08031	0.999

Rheological comparison of V2A2E3-derived peptide (Figure 4E/S4): storage modulus					
**condition 1**	**condition 2**	**estimate**	**conf. low**	**conf. high**	**adj.p.value**
V2A2E2 -UV	PEG only (no DA-PA)	3895	-48860	56650	1.000E+00
V2A2E2 +UV	PEG only (no DA-PA)	37372	-15383	90127	3.387E-01
V2A2E3-Click -UV	PEG only (no DA-PA)	56918	4163	109673	2.736E-02
V2A2E3-Click +UV	PEG only (no DA-PA)	725552	672797	778307	2.330E-14
V2A2E3 -UV	PEG only (no DA-PA)	7616	-45139	60371	1.000E+00

V2A2E3 +UV	PEG only (no DA-PA)	575618	522863	628373	2.330E-14
V2A2E4 -UV	PEG only (no DA-PA)	8792	-43963	61547	9.999E-01
V2A2E4 +UV	PEG only (no DA-PA)	46928	-5827	99683	1.108E-01
V3A3E3 -UV	PEG only (no DA-PA)	15598	-37157	68353	9.901E-01
V3A3E3 +UV	PEG only (no DA-PA)	54962	2207	107717	3.643E-02
V2A2E2 +UV	V2A2E2 -UV	33477	-19278	86232	4.854E-01
V2A2E3-Click -UV	V2A2E2 -UV	53023	268	105778	4.813E-02
V2A2E3-Click +UV	V2A2E2 -UV	721657	668902	774412	2.330E-14
V2A2E3 -UV	V2A2E2 -UV	3721	-49034	56476	1.000E+00
V2A2E3 +UV	V2A2E2 -UV	571723	518968	624478	2.330E-14
V2A2E4 -UV	V2A2E2 -UV	4897	-47858	57652	1.000E+00
V2A2E4 +UV	V2A2E2 -UV	43033	-9722	95788	1.807E-01
V3A3E3 -UV	V2A2E2 -UV	11703	-41052	64458	9.990E-01
V3A3E3 +UV	V2A2E2 -UV	51067	-1688	103822	6.338E-02
V2A2E3-Click -UV	V2A2E2 +UV	19547	-33208	72302	9.541E-01
V2A2E3-Click +UV	V2A2E2 +UV	688180	635425	740935	2.330E-14
V2A2E3 -UV	V2A2E2 +UV	-29756	-82511	22999	6.408E-01
V2A2E3 +UV	V2A2E2 +UV	538247	485492	591002	2.330E-14
V2A2E4 -UV	V2A2E2 +UV	-28580	-81335	24175	6.894E-01
V2A2E4 +UV	V2A2E2 +UV	9557	-43198	62312	9.998E-01
V3A3E3 -UV	V2A2E2 +UV	-21773	-74528	30982	9.131E-01
V3A3E3 +UV	V2A2E2 +UV	17590	-35165	70345	9.769E-01
V2A2E3-Click +UV	V2A2E3-Click -UV	668633	615878	721388	2.330E-14
V2A2E3 -UV	V2A2E3-Click -UV	-49303	-102058	3452	8.077E-02
V2A2E3 +UV	V2A2E3-Click -UV	518700	465945	571455	2.330E-14
V2A2E4 -UV	V2A2E3-Click -UV	-48127	-100882	4628	9.461E-02
V2A2E4 +UV	V2A2E3-Click -UV	-9990	-62745	42765	9.997E-01
V3A3E3 -UV	V2A2E3-Click -UV	-41320	-94075	11435	2.211E-01
V3A3E3 +UV	V2A2E3-Click -UV	-1957	-54712	50798	1.000E+00
V2A2E3 -UV	V2A2E3-Click +UV	-717936	-770691	-665181	2.330E-14

V2A2E3 +UV	V2A2E3-Click +UV	-149933	-202688	-97178	4.340E-08
V2A2E4 -UV	V2A2E3-Click +UV	-716760	-769515	-664005	2.330E-14
V2A2E4 +UV	V2A2E3-Click +UV	-678623	-731378	-625868	2.330E-14
V3A3E3 -UV	V2A2E3-Click +UV	-709953	-762708	-657198	2.330E-14
V3A3E3 +UV	V2A2E3-Click +UV	-670590	-723345	-617835	2.330E-14
V2A2E3 +UV	V2A2E3 -UV	568003	515248	620758	2.330E-14
V2A2E4 -UV	V2A2E3 -UV	1176	-51579	53931	1.000E+00
V2A2E4 +UV	V2A2E3 -UV	39313	-13442	92068	2.766E-01
V3A3E3 -UV	V2A2E3 -UV	7983	-44772	60738	1.000E+00
V3A3E3 +UV	V2A2E3 -UV	47346	-5409	100101	1.049E-01
V2A2E4 -UV	V2A2E3 +UV	-566827	-619582	-514072	2.330E-14
V2A2E4 +UV	V2A2E3 +UV	-528690	-581445	-475935	2.330E-14
V3A3E3 -UV	V2A2E3 +UV	-560020	-612775	-507265	2.330E-14
V3A3E3 +UV	V2A2E3 +UV	-520657	-573412	-467902	2.330E-14
V2A2E4 +UV	V2A2E4 -UV	38137	-14618	90892	3.133E-01
V3A3E3 -UV	V2A2E4 -UV	6807	-45948	59562	1.000E+00
V3A3E3 +UV	V2A2E4 -UV	46170	-6585	98925	1.223E-01
V3A3E3 -UV	V2A2E4 +UV	-31330	-84085	21425	5.746E-01
V3A3E3 +UV	V2A2E4 +UV	8033	-44722	60788	1.000E+00
V3A3E3 +UV	V3A3E3 -UV	39363	-13392	92118	2.751E-01

Rheological comparison of V2A2E3-derived peptide (Figure 4E/S4): loss modulus					
**condition 1**	**condition 2**	**estimate**	**conf. low**	**conf. high**	**adj.p.value**
V2A2E2 -UV	PEG only (no DA-PA)	947	-13210	15105	1.000E+00
V2A2E2 +UV	PEG only (no DA-PA)	11525	-2633	25682	1.827E-01
V2A2E3-Click -UV	PEG only (no DA-PA)	7374	-6783	21532	7.334E-01
V2A2E3-Click +UV	PEG only (no DA-PA)	165081	150924	179239	2.330E-14
V2A2E3 -UV	PEG only (no DA-PA)	1310	-12848	15467	1.000E+00

V2A2E3 +UV	PEG only (no DA-PA)	132881	118724	147039	2.330E-14
V2A2E4 -UV	PEG only (no DA-PA)	1560	-12598	15717	1.000E+00
V2A2E4 +UV	PEG only (no DA-PA)	10785	-3373	24942	2.513E-01
V3A3E3 -UV	PEG only (no DA-PA)	2747	-11411	16904	9.997E-01
V3A3E3 +UV	PEG only (no DA-PA)	13098	-1060	27255	8.637E-02
V2A2E2 +UV	V2A2E2 -UV	10578	-3580	24735	2.735E-01
V2A2E3-Click -UV	V2A2E2 -UV	6427	-7730	20584	8.564E-01
V2A2E3-Click +UV	V2A2E2 -UV	164134	149977	178291	2.330E-14
V2A2E3 -UV	V2A2E2 -UV	362	-13795	14520	1.000E+00
V2A2E3 +UV	V2A2E2 -UV	131934	117777	146091	2.330E-14
V2A2E4 -UV	V2A2E2 -UV	613	-13545	14770	1.000E+00
V2A2E4 +UV	V2A2E2 -UV	9837	-4320	23995	3.636E-01
V3A3E3 -UV	V2A2E2 -UV	1800	-12358	15957	1.000E+00
V3A3E3 +UV	V2A2E2 -UV	12151	-2007	26308	1.370E-01
V2A2E3-Click -UV	V2A2E2 +UV	-4151	-18308	10007	9.907E-01
V2A2E3-Click +UV	V2A2E2 +UV	153556	139399	167714	2.330E-14
V2A2E3 -UV	V2A2E2 +UV	-10215	-24373	3942	3.156E-01
V2A2E3 +UV	V2A2E2 +UV	121356	107199	135514	2.330E-14
V2A2E4 -UV	V2A2E2 +UV	-9965	-24122	4192	3.469E-01
V2A2E4 +UV	V2A2E2 +UV	-740	-14898	13417	1.000E+00
V3A3E3 -UV	V2A2E2 +UV	-8778	-22935	5379	5.169E-01
V3A3E3 +UV	V2A2E2 +UV	1573	-12584	15730	1.000E+00
V2A2E3-Click +UV	V2A2E3-Click -UV	157707	143550	171864	2.330E-14
V2A2E3 -UV	V2A2E3-Click -UV	-6065	-20222	8093	8.935E-01
V2A2E3 +UV	V2A2E3-Click -UV	125507	111350	139664	2.330E-14
V2A2E4 -UV	V2A2E3-Click -UV	-5814	-19972	8343	9.155E-01
V2A2E4 +UV	V2A2E3-Click -UV	3410	-10747	17568	9.980E-01
V3A3E3 -UV	V2A2E3-Click -UV	-4627	-18785	9530	9.798E-01
V3A3E3 +UV	V2A2E3-Click -UV	5724	-8434	19881	9.227E-01
V2A2E3 -UV	V2A2E3-Click +UV	-163772	-177929	-149614	2.330E-14

V2A2E3 +UV	V2A2E3-Click +UV	-32200	-46357	-18043	2.090E-06
V2A2E4 -UV	V2A2E3-Click +UV	-163521	-177679	-149364	2.330E-14
V2A2E4 +UV	V2A2E3-Click +UV	-154297	-168454	-140139	2.330E-14
V3A3E3 -UV	V2A2E3-Click +UV	-162334	-176492	-148177	2.330E-14
V3A3E3 +UV	V2A2E3-Click +UV	-151983	-166141	-137826	2.330E-14
V2A2E3 +UV	V2A2E3 -UV	131572	117414	145729	2.330E-14
V2A2E4 -UV	V2A2E3 -UV	250	-13907	14408	1.000E+00
V2A2E4 +UV	V2A2E3 -UV	9475	-4682	23632	4.133E-01
V3A3E3 -UV	V2A2E3 -UV	1437	-12720	15595	1.000E+00
V3A3E3 +UV	V2A2E3 -UV	11788	-2369	25946	1.621E-01
V2A2E4 -UV	V2A2E3 +UV	-131321	-145479	-117164	2.330E-14
V2A2E4 +UV	V2A2E3 +UV	-122097	-136254	-107939	2.330E-14
V3A3E3 -UV	V2A2E3 +UV	-130134	-144292	-115977	2.330E-14
V3A3E3 +UV	V2A2E3 +UV	-119783	-133941	-105626	2.330E-14
V2A2E4 +UV	V2A2E4 -UV	9225	-4933	23382	4.495E-01
V3A3E3 -UV	V2A2E4 -UV	1187	-12970	15344	1.000E+00
V3A3E3 +UV	V2A2E4 -UV	11538	-2619	25695	1.816E-01
V3A3E3 -UV	V2A2E4 +UV	-8038	-22195	6120	6.327E-01
V3A3E3 +UV	V2A2E4 +UV	2313	-11844	16471	9.999E-01
V3A3E3 +UV	V3A3E3 -UV	10351	-3806	24508	2.994E-01

Rheological comparison of V2A2E3-derived peptide (Figure 4E/S4): tan δ					
**condition 1**	**condition 2**	**esti mate**	**conf. low**	**conf. high**	**adj.p.value**
V2A2E2 -UV	PEG only (no DA-PA)	0.117	0.050	0.184	0.00010997
V2A2E2 +UV	PEG only (no DA-PA)	0.242	0.175	0.308	4.41E-10
V2A2E3-Click -UV	PEG only (no DA-PA)	0.083	0.016	0.150	0.0072026
V2A2E3-Click +UV	PEG only (no DA-PA)	0.184	0.118	0.251	7.23E-08

V2A2E3 -UV	PEG only (no DA-PA)	0.095	0.028	0.161	0.00173925
V2A2E3 +UV	PEG only (no DA-PA)	0.187	0.120	0.254	5.45E-08
V2A2E4 -UV	PEG only (no DA-PA)	0.103	0.036	0.169	0.00065129
V2A2E4 +UV	PEG only (no DA-PA)	0.177	0.110	0.243	1.51E-07
V3A3E3 -UV	PEG only (no DA-PA)	0.113	0.046	0.180	0.00018013
V3A3E3 +UV	PEG only (no DA-PA)	0.187	0.120	0.253	5.63E-08
V2A2E2 +UV	V2A2E2 -UV	0.124	0.058	0.191	4.63E-05
V2A2E3-Click -UV	V2A2E2 -UV	-0.034	-0.101	0.033	0.75271489
V2A2E3-Click +UV	V2A2E2 -UV	0.067	0.000	0.134	0.0479266
V2A2E3 -UV	V2A2E2 -UV	-0.023	-0.089	0.044	0.97453283
V2A2E3 +UV	V2A2E2 -UV	0.070	0.003	0.137	0.03437412
V2A2E4 -UV	V2A2E2 -UV	-0.015	-0.081	0.052	0.99908938
V2A2E4 +UV	V2A2E2 -UV	0.060	-0.007	0.126	0.10735551
V3A3E3 -UV	V2A2E2 -UV	-0.004	-0.071	0.063	0.99999999
V3A3E3 +UV	V2A2E2 -UV	0.070	0.003	0.136	0.03572781
V2A2E3-Click -UV	V2A2E2 +UV	-0.159	-0.225	-0.092	9.98E-07
V2A2E3-Click +UV	V2A2E2 +UV	-0.057	-0.124	0.009	0.13495975
V2A2E3 -UV	V2A2E2 +UV	-0.147	-0.214	-0.080	3.50E-06
V2A2E3 +UV	V2A2E2 +UV	-0.054	-0.121	0.012	0.17955131
V2A2E4 -UV	V2A2E2 +UV	-0.139	-0.206	-0.072	8.53E-06
V2A2E4 +UV	V2A2E2 +UV	-0.065	-0.131	0.002	0.06153499
V3A3E3 -UV	V2A2E2 +UV	-0.129	-0.195	-0.062	2.87E-05
V3A3E3 +UV	V2A2E2 +UV	-0.055	-0.121	0.012	0.17388028
V2A2E3-Click +UV	V2A2E3-Click -UV	0.101	0.034	0.168	0.00077724
V2A2E3 -UV	V2A2E3-Click -UV	0.012	-0.055	0.078	0.99988222
V2A2E3 +UV	V2A2E3-Click -UV	0.104	0.037	0.171	0.0005427
V2A2E4 -UV	V2A2E3-Click -UV	0.019	-0.047	0.086	0.99088831
V2A2E4 +UV	V2A2E3-Click -UV	0.094	0.027	0.160	0.00194707
V3A3E3 -UV	V2A2E3-Click -UV	0.030	-0.037	0.097	0.86240225
V3A3E3 +UV	V2A2E3-Click -UV	0.104	0.037	0.170	0.00056557
V2A2E3 -UV	V2A2E3-Click +UV	-0.090	-0.156	-0.023	0.00323412
V2A2E3 +UV	V2A2E3-Click +UV	0.003	-0.064	0.070	1

V2A2E4 -UV	V2A2E3-Click +UV	-0.082	-0.148	-0.015	0.0085783
V2A2E4 +UV	V2A2E3-Click +UV	-0.007	-0.074	0.059	0.99999804
V3A3E3 -UV	V2A2E3-Click +UV	-0.071	-0.138	-0.004	0.03002771
V3A3E3 +UV	V2A2E3-Click +UV	0.003	-0.064	0.069	1
V2A2E3 +UV	V2A2E3 -UV	0.093	0.026	0.159	0.00225609
V2A2E4 -UV	V2A2E3 -UV	0.008	-0.059	0.075	0.99999626
V2A2E4 +UV	V2A2E3 -UV	0.082	0.015	0.149	0.00805077
V3A3E3 -UV	V2A2E3 -UV	0.018	-0.048	0.085	0.99395108
V3A3E3 +UV	V2A2E3 -UV	0.092	0.026	0.159	0.00235163
V2A2E4 -UV	V2A2E3 +UV	-0.085	-0.151	-0.018	0.00600874
V2A2E4 +UV	V2A2E3 +UV	-0.010	-0.077	0.056	0.99995619
V3A3E3 -UV	V2A2E3 +UV	-0.074	-0.141	-0.007	0.02133762
V3A3E3 +UV	V2A2E3 +UV	-0.000	-0.067	0.066	1
V2A2E4 +UV	V2A2E4 -UV	0.074	0.008	0.141	0.0209438
V3A3E3 -UV	V2A2E4 -UV	0.011	-0.056	0.077	0.99994969
V3A3E3 +UV	V2A2E4 -UV	0.084	0.018	0.151	0.00626079
V3A3E3 -UV	V2A2E4 +UV	-0.064	-0.130	0.003	0.06947609
V3A3E3 +UV	V2A2E4 +UV	0.010	-0.057	0.077	0.99996764
V3A3E3 +UV	V3A3E3 -UV	0.074	0.007	0.140	0.02219971

PEG crosslinker comparison PEG only no PA (Figure 6): storage modulus

condition 1	condition 2	estimate	conf.low	conf.high	adj.p.value
K-PanMMP crosslinked	E-PanMMP crosslinked	1708.80	1490.14	1927.46	1.01E-06
PEG crosslinked	E-PanMMP crosslinked	-398.80	-617.46	-180.14	0.003345202
PEG crosslinked	K-PanMMP crosslinked	-2107.60	-2326.26	-1888.94	4.04E-07

PEG crosslinker comparison PEG only no PA (Figure 6): loss modulus

condition 1	condition 2	estimate	conf.low	conf.high	adj.p.value
K-PanMMP crosslinked	E-PanMMP crosslinked	85.69	-2.18	173.56	0.054924149
PEG crosslinked	E-PanMMP crosslinked	102.34	14.48	190.21	0.027235185

PEG crosslinked	K-PanMMP crosslinked	16.65	-71.21	104.52	0.834668405

PEG crosslinker comparison PEG only no PA (Figure 6): tan δ

condition 1	condition 2	estimate	conf.low	conf.high	adj.p.value
K-PanMMP crosslinked	E-PanMMP crosslinked	-11930	-15311.21677	-8548.78323[illegible]	9.07E-05
PEG crosslinked	E-PanMMP crosslinked	-14449.3333[illegible]	-17830.5501	-11068.11657	2.98E-05
PEG crosslinked	K-PanMMP crosslinked	-2519.33333[illegible]	-5900.5501	861.8834338	0.134105519

PEG crosslinker comparison before UV exposure (Figure 6): storage modulus

condition 1	condition 2	estimate	conf.low	conf.high	adj.p.value
PEG crosslinked	E-PanMMP crosslinked	-11930.00	-15311.22	-8548.78	9.07E-05
K-PanMMP crosslinked	E-PanMMP crosslinked	-14449.33	-17830.55	-11068.12	2.98E-05
K-PanMMP crosslinked	PEG crosslinked	-2519.33	-5900.55	861.88	0.134105519

PEG crosslinker comparison before UV exposure (Figure 6): loss modulus

condition 1	condition 2	estimate	conf.low	conf.high	adj.p.value
PEG crosslinked	E-PanMMP crosslinked	-3034.53	-4090.60	-1978.47	0.00029048
K-PanMMP crosslinked	E-PanMMP crosslinked	-2751.00	-3807.06	-1694.94	0.000501135
K-PanMMP crosslinked	PEG crosslinked	283.53	-772.53	1339.60	0.703265329

PEG crosslinker comparison before UV exposure (Figure 6): tan δ

condition 1	condition 2	estimate	conf.low	conf.high	adj.p.value
PEG crosslinked	E-PanMMP_no_UV	-0.080	-0.117	-0.043	0.001378852
K-PanMMP crosslinked	E-PanMMP_no_UV	-0.030	-0.068	0.007	0.100402546
K-PanMMP crosslinked	PEG crosslinked	0.050	0.013	0.087	0.014661944

PEG crosslinker comparison after UV exposure (Figure 6): storage modulus

condition 1	condition 2	estimate	conf.low	conf.high	adj.p.value
PEG crosslinked	E-PanMMP crosslinked	-60203.33	-136426.08	16019.41	0.112547532
K-PanMMP crosslinked	E-PanMMP crosslinked	459866.67	383643.92	536089.41	3.61E-06
K-PanMMP crosslinked	PEG crosslinked	520070.00	443847.26	596292.74	1.84E-06

PEG crosslinker comparison after UV exposure (Figure 6): loss modulus

condition 1	condition 2	estimate	conf.low	conf.high	adj.p.value
PEG crosslinked	E-PanMMP crosslinked	-7428.67	-27981.11	13123.78	0.543448598
K-PanMMP crosslinked	E-PanMMP crosslinked	115616.67	95064.22	136169.11	5.54E-06
K-PanMMP crosslinked	PEG crosslinked	123045.33	102492.89	143597.78	3.78E-06

PEG crosslinker comparison after UV exposure (Figure 6): tan δ

condition 1	condition 2	estimate	conf.low	conf.high	adj.p.value
PEG crosslinked	E-PanMMP crosslinked	0.027	-0.013	0.066	0.172185461
K-PanMMP crosslinked	E-PanMMP crosslinked	0.083	0.044	0.122	0.001565901
K-PanMMP crosslinked	PEG crosslinked	0.056	0.017	0.095	0.011002327

Polymer comparison (Figure 7): storage modulus

condition 1	condition 2	estimate	conf.low	conf.high	adj.p.value
Alginate + V2A2E3 -UV	Alginate	11113.67	-46540.07	68767.40	0.984602141
Alginate + V2A2E3 +UV	Alginate	128557.00	70903.26	186210.74	8.36E-05
PEG (K-PanMMP crosslinked)	Alginate	-4194.67	-61848.40	53459.07	0.999845512
PEG + V2A2E3 -UV	Alginate	3421.00	-54232.74	61074.74	0.999943384

PEG + V2A2E3 +UV	Alginate	571423.67	513769.93	629077.40	3.27E-12
Alginate + V2A2E3 +UV	Alginate + V2A2E3 -UV	117443.33	59789.60	175097.07	0.000201433
PEG (K-PanMMP crosslinked)	Alginate + V2A2E3 -UV	-15308.33	-72962.07	42345.40	0.941338975
PEG + V2A2E3 -UV	Alginate + V2A2E3 -UV	-7692.67	-65346.40	49961.07	0.997124011
PEG + V2A2E3 +UV	Alginate + V2A2E3 -UV	560310.00	502656.26	617963.74	3.68E-12
PEG (K-PanMMP crosslinked)	Alginate + V2A2E3 +UV	-132751.67	-190405.40	-75097.93	6.07E-05
PEG + V2A2E3 -UV	Alginate + V2A2E3 +UV	-125136.00	-182789.74	-67482.26	0.000109023
PEG + V2A2E3 +UV	Alginate + V2A2E3 +UV	442866.67	385212.93	500520.40	5.12E-11
PEG + V2A2E3 -UV	PEG (K-PanMMP crosslinked)	7615.67	-50038.07	65269.40	0.997256805
PEG + V2A2E3 +UV	PEG (K-PanMMP crosslinked)	575618.33	517964.60	633272.07	3.12E-12
PEG + V2A2E3 +UV	PEG + V2A2E3 -UV	568002.67	510348.93	625656.40	3.39E-12

Polymer comparison (Figure 7): loss modulus

condition 1	condition 2	estimate	conf.low	conf.high	adj.p.value
Alginate + V2A2E3 -UV	Alginate	2580.67	-13221.67	18383.00	0.992670808
Alginate + V2A2E3 +UV	Alginate	32156.00	16353.67	47958.33	0.000203481
PEG (K-PanMMP crosslinked)	Alginate	-1641.85	-17444.19	14160.48	0.999125468
PEG + V2A2E3 -UV	Alginate	-332.33	-16134.67	15470.00	0.999999675
PEG + V2A2E3 +UV	Alginate	131239.33	115437.00	147041.67	1.54E-11
Alginate + V2A2E3 +UV	Alginate + V2A2E3 -UV	29575.33	13773.00	45377.67	0.000445897
PEG (K-PanMMP crosslinked)	Alginate + V2A2E3 -UV	-4222.52	-20024.85	11579.81	0.93986578
PEG + V2A2E3 -UV	Alginate + V2A2E3 -UV	-2913.00	-18715.33	12889.33	0.987360319
PEG + V2A2E3 +UV	Alginate + V2A2E3 -UV	128658.67	112856.33	144461.00	2.05E-11
PEG (K-PanMMP crosslinked)	Alginate + V2A2E3 +UV	-33797.85	-49600.19	-17995.52	0.000125866

PEG + V2A2E3 -UV	Alginate + V2A2E3 +UV	-32488.33	-48290.67	-16686.00	0.000184413
PEG + V2A2E3 +UV	Alginate + V2A2E3 +UV	99083.33	83281.00	114885.67	1.04E-09
PEG + V2A2E3 -UV	PEG (K-PanMMP crosslinked)	1309.52	-14492.81	17111.85	0.999707913
PEG + V2A2E3 +UV	PEG (K-PanMMP crosslinked)	132881.19	117078.85	148683.52	1.30E-11
PEG + V2A2E3 +UV	PEG + V2A2E3 -UV	131571.67	115769.33	147374.00	1.49E-11

Polymer comparison (Figure 7): tan δ

condition 1	condition 2	estimate	conf.low	conf.high	adj.p.value
Alginate + V2A2E3 -UV	Alginate	-0.018	-0.098	0.061	0.96579916
Alginate + V2A2E3 +UV	Alginate	-0.007	-0.086	0.073	0.999687227
PEG (K-PanMMP crosslinked)	Alginate	-0.215	-0.295	-0.136	1.12E-05
PEG + V2A2E3 -UV	Alginate	-0.121	-0.200	-0.042	0.002685388
PEG + V2A2E3 +UV	Alginate	-0.028	-0.108	0.051	0.829003684
Alginate + V2A2E3 +UV	Alginate + V2A2E3 -UV	0.012	-0.068	0.091	0.995295384
PEG (K-PanMMP crosslinked)	Alginate + V2A2E3 -UV	-0.197	-0.276	-0.118	2.83E-05
PEG + V2A2E3 -UV	Alginate + V2A2E3 -UV	-0.102	-0.182	-0.023	0.00964923
PEG + V2A2E3 +UV	Alginate + V2A2E3 -UV	-0.010	-0.089	0.069	0.997898516
PEG (K-PanMMP crosslinked)	Alginate + V2A2E3 +UV	-0.209	-0.288	-0.129	1.55E-05
PEG + V2A2E3 -UV	Alginate + V2A2E3 +UV	-0.114	-0.194	-0.035	0.004237744
PEG + V2A2E3 +UV	Alginate + V2A2E3 +UV	-0.022	-0.101	0.058	0.934523323
PEG + V2A2E3 -UV	PEG (K-PanMMP crosslinked)	0.095	0.015	0.174	0.016805035
PEG + V2A2E3 +UV	PEG (K-PanMMP crosslinked)	0.187	0.108	0.266	4.78E-05
PEG + V2A2E3 +UV	PEG + V2A2E3 -UV	0.093	0.013	0.172	0.019533422
